# Crowdsourcing Language Change with Smartphone Applications

**DOI:** 10.1371/journal.pone.0143060

**Published:** 2016-01-04

**Authors:** Adrian Leemann, Marie-José Kolly, Ross Purves, David Britain, Elvira Glaser

**Affiliations:** 1 Phonetics Laboratory, Department of Theoretical and Applied Linguistics, University of Cambridge, Cambridge, United Kingdom; 2 Laboratoire d’Informatique pour la Mécanique et les Sciences de l’Ingénieur, CNRS, Orsay, France; 3 Department of Comparative Linguistics, University of Zurich, Zurich, Switzerland; 4 Department of Geography, University of Zurich, Zurich, Switzerland; 5 Department of English, University of Bern, Bern, Switzerland; 6 German Department, University of Zurich, Zurich, Switzerland; Beihang University, CHINA

## Abstract

Crowdsourcing linguistic phenomena with smartphone applications is relatively new. In linguistics, apps have predominantly been developed to create pronunciation dictionaries, to train acoustic models, and to archive endangered languages. This paper presents the first account of how apps can be used to collect data suitable for documenting language change: we created an app, Dialäkt Äpp (DÄ), which predicts users’ dialects. For 16 linguistic variables, users select a dialectal variant from a drop-down menu. DÄ then geographically locates the user’s dialect by suggesting a list of communes where dialect variants most similar to their choices are used. Underlying this prediction are 16 maps from the historical Linguistic Atlas of German-speaking Switzerland, which documents the linguistic situation around 1950. Where users disagree with the prediction, they can indicate what they consider to be their dialect’s location. With this information, the 16 variables can be assessed for language change. Thanks to the playfulness of its functionality, DÄ has reached many users; our linguistic analyses are based on data from nearly 60,000 speakers. Results reveal a relative stability for phonetic variables, while lexical and morphological variables seem more prone to change. Crowdsourcing large amounts of dialect data with smartphone apps has the potential to complement existing data collection techniques and to provide evidence that traditional methods cannot, with normal resources, hope to gather. Nonetheless, it is important to emphasize a range of methodological caveats, including sparse knowledge of users’ linguistic backgrounds (users only indicate age, sex) and users’ self-declaration of their dialect. These are discussed and evaluated in detail here. Findings remain intriguing nevertheless: as a means of quality control, we report that traditional dialectological methods have revealed trends similar to those found by the app. This underlines the validity of the crowdsourcing method. We are presently extending DÄ architecture to other languages.

## Introduction

Crowdsourcing, “the practice of obtaining needed […] content by soliciting contributions from a large group of people and especially from the online community […],” powerfully capitalizes on the fact that *none of us is as smart as all of us* [1]. Crowdsourcing is not a new scientific phenomenon. In ornithology, for example, the North American “Christmas Bird Count” is now in its 115^th^ year. Since 1900 the project has encouraged bird enthusiasts to count and record the number of birds of each species they witness on Christmas Day. In 2012, more than 70,000 people participated in this crowdsourcing project [2]. In linguistics, one of the first accounts of collecting dialect data in a crowdsourcing fashion was the German dialect survey conducted by Georg Wenker. Wenker began documenting dialects in the late 19^th^ century by distributing some 50,000 questionnaires with 40 test sentences to schoolmasters across Germany, achieving a 90% response rate. The survey and responses were written in Standard German orthography as well as localized transcriptions and were collated, stored, and prepared for display on large paper maps [3]. A cartographical structure for mapping dialectological evidence was born [4]. A century and a half later, paper is being replaced by online surveys and smartphone applications (apps) as a very powerful and flexible medium for crowdsourcing language data.

Internet-based crowdsourcing has recently emerged as a means of collecting language data in speech science [5]. Amazon’s Mechanical Turk [6], a key player in current crowdsourcing platforms, has been used extensively in recent years to collect data to develop the capabilities of human language technologies (for an overview paper see [7]). Labor that cannot yet be conducted by computers is crowdsourced via so-called ‘Turkers’. In natural language processing, this work principally involves the creation of speech and text annotation. Crowdsourcing data through mobile devices to study linguistic phenomena is even more recent. This is surprising given both the increasing integration of voice-operated technology in mobile communication and the burgeoning number of mobile apps. Used as a medium for rapid, large-scale data collection, exciting opportunities arise for a wide range of cognate disciplines [8]. Smartphones are ubiquitous, unobtrusive, and computationally powerful, offering vast potential for gathering data on the real-world behaviors of millions of people without requiring subjects to come into a lab [9]. Smartphone apps have been used to create pronunciation dictionaries [10], collect speech as a means to train acoustic models for automatic speech recognition [11], document endangered languages [12] and gather grammaticality judgments [13]. A more passive form of crowdsourcing, with subjects unaware of the scientific use of their data, is large-scale analysis of diatopic language variation using geotagged Twitter posts; one such study that used lexical parameters reports that the Spanish language is split into two superdialects, namely an urban variety used across major American and Spanish cities and a diverse form that encompasses rural areas and small towns [14].

In this paper we demonstrate how crowdsourcing speech data with the smartphone app ‘Dialäkt Äpp’ (hereafter DÄ; [15]) allows documentation of language change and areal variation. DÄ’s main function is the prediction of a user’s home dialect location based on a 16-question survey pre-determined by phoneticians. Following the prediction, users can evaluate the result and indicate their real dialect location. Underlying the 16 questions are 16 maps from the *Linguistic Atlas of German-speaking Switzerland* (*Sprachatlas der Deutschen Schweiz*)–hereafter referred to as the *Atlas*—which documents the linguistic situation in German-speaking Switzerland roughly 70 years ago in 560 localities [16]. Whether for educational or entertainment purposes, DÄ has been downloaded nearly 80,000 times. Feedback from nearly 60,000 users provides a contemporary snapshot of the Swiss German dialect landscape, which can be used to investigate diachronic variation by comparison with *Atlas* data [17, 18, 19]. Upon its release, DÄ became the most downloaded free iPhone app in Switzerland [17]. The app also received broad media attention in German-speaking Europe: it remained in the top three German educational apps in Switzerland for several months and it was covered by *20 Minuten*, the most popular Swiss daily. This attention is important, as it is an essential part in the success of crowdsourced projects.

The potential to predict someone’s dialect location with such a tool has caught the public’s interest not only in German-speaking Switzerland but also in the United States. Half a year after the release of DÄ, the *New York Times* published an online app—the ‘Dialect quiz’ [20]–that predicts the user’s American English dialect. The quiz consists of 25 questions such as, “What is your generic term for a sweetened carbonated beverage?”. The user provides their answer (*pop*, *soda*, *coke*, or *other*) and proceeds to the next question. In the end, dialect location predictions are displayed. The statistics used to determine the prediction of dialect location are pre-calculated from self-reported responses in the Harvard Dialect Survey [21] in conjunction with a supplementary survey of 350,000 people [22]. Though posted on the *Times* website within the last 10 days of 2013, this quiz became the year’s most popular piece of content [23]. This is no small accomplishment: the *Times* is the most popular news website in America, accessed by over 30 million unique users per month.

Previous research on Swiss German dialects using crowdsourced data is based on web surveys and is comparatively scant. The largest sample previously obtained in this way for Swiss German dialect data includes 14,000 speakers [24]. [25] crowdsourced lexical dialect data from more than 5,000 speakers and compared her results to those of the *Atlas*, finding a striking convergence towards Standard German. She further hypothesized that phonetic variables as elicited in the *Atlas* may have undergone less change than lexical variables (a finding reported in [26]). [25] further revealed, unsurprisingly, that younger speakers were more likely to deviate from the *Atlas* than older speakers (cf. [27]).

In the present paper, we demonstrate that data collected through DÄ and subsequent diachronic analyses have the potential to shed considerable light on dialectal and linguistic diversity. In section 2 of this paper, we introduce how the app works and how we have applied it to the analysis of language change in German-speaking Switzerland. Section 3 shows results based on data elicited through DÄ, that are then discussed in section 4. A major part of the discussion is dedicated to highlighting the innovative nature of this approach to data collection and analysis while critically reflecting upon it as a dialect research methodology. Section 5 introduces the quality control we applied on the results presented.

## Methods

The primary function of DÄ is the prediction of a user’s dialect location. This is based on 16 discriminative maps of different linguistic variables, from [16]. Here, we discuss the criteria according to which we selected the variables (2.1), the implementation of the prediction algorithm on a mobile platform (2.2), and how language change can be documented with the data collected (2.3). An extensive description of DÄ’s further functionalities and the methods for implementing them is given in [17].

### 2.1 Variable selection

**Fig 1:**
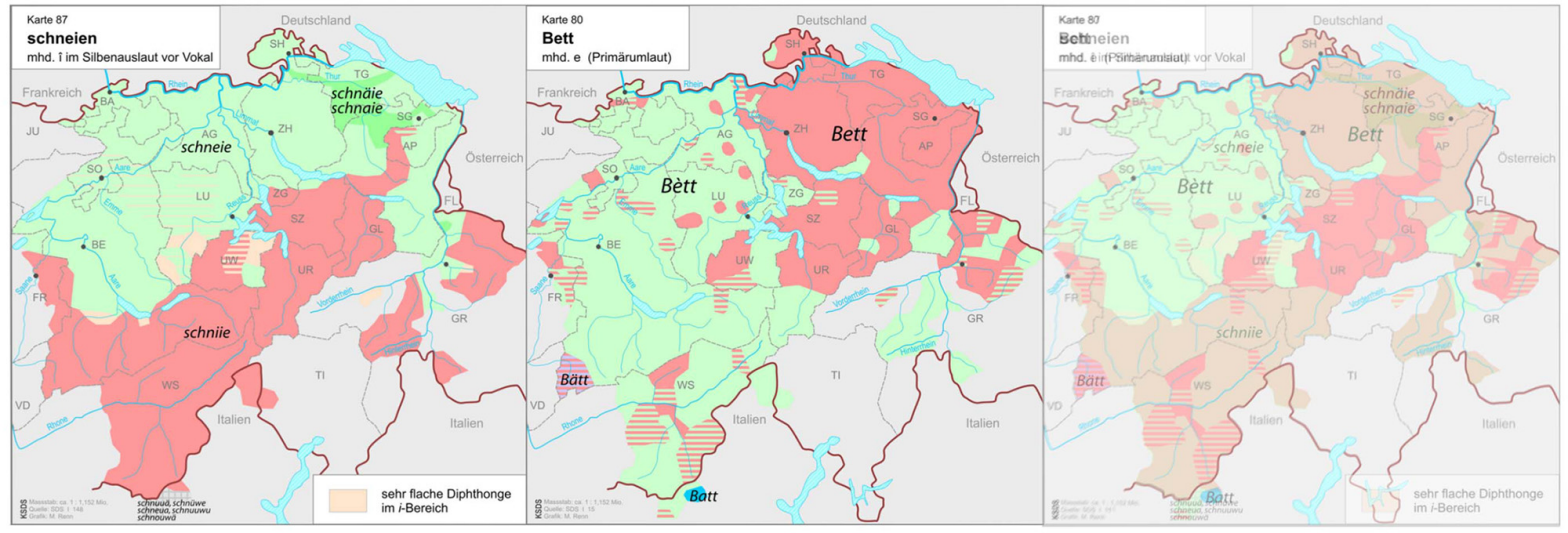
Variants of *schneien* ‘to snow’ (left), *Bett* ‘bed’ (center), and the four-quadrant intersection of the two maps (right; maps adapted from [28]).

Since the *Atlas* documented dialects spoken by mostly older people (the most common dialectological approach at the time) around 70 years ago, we selected linguistic variables that we assumed to still have relatively stable geographical distributions in order to get dialect prediction results as precise as possible. Since previous research had shown that the isoglosses of some lexical variables had undergone major changes [25], we primarily selected phonetic variables, i.e. maps (see Table 1). Variables each showing different geographical distributions from the other were chosen so that very small areas could be distinguished from each other on the basis of a unique combination of variants across the set of variables. Overlaying only two variables with two variants each, for example, partitions the linguistic area into four quadrants (Fig 1, right): the variants for *schneien* ‘to snow’, for example, creates a northern and a southern area (Fig 1, left), while the variants for *Bett* ‘bed’ splits Swiss German dialects into western and eastern variants (Fig 1, center).

**Table 1:** Variables chosen for dialect prediction (MHG = Middle High German) according to [29], examples, numbers, and types of Swiss German variables; adapted from [17].

Standard German word	Variable	Examples of variants	N	Type
*Abend* ‘evening’	MHG -*ent*, *-ant*, *-int*, *-unt*	*Aab**e*, *Aab**ig*	13	phon.
*Abend* ‘evening’	MHG *â*, *ô*	*Aa**be* [aː], *Aa**be* [ɑː]	8	phon.
*Apfelüberrest* ‘apple core’	lexeme	*Bütschgi*, *Gröibschi*	39	lex.
*Augen* ‘eyes’	MHG *ou*	*Ai**ge*, *Ou**ge*	11	phon.
*Bett* ‘bed’	MHG *e*	*B**e**tt* [e], *B**e**tt* [ɛ]	2	phon.
*Donnerstag* ‘Thursday’	MHG *o*, *u*	*D**o**nschtig* [o], *D**u**nschtig* [u]	8	phon.
*Kelle* ‘ladle’	MHG *ll*	*Chä**l**e*, *Chä**u**e*	5	phon.
*Kind* ‘child’	MHG *k*, *ch*	*Ch**ind* [x], *Sch**ind* [ʃ]	4	phon.
*Tanne* ‘fir tree’	MHG *n*, *nn*	*Ta**n**e*, *Ta**nn**e*	2	phon.
*fragen* ‘to ask’	MHG *â*, *ê*, *ô*	*fr**aa**ge*, *fr**ää**ge*	10	phon.
*heben* ‘to lift’	MHG *u*	*l**u**pfe* [u], [ʊ], *l**ü**pfe* [y], [ʏ]	3	phon.
*hinauf* ‘upwards’	MHG *hin-ûf*	*embruf/embrüf*, *uehi*	31	morph.
*schneien* ‘to snow’	MHG *î*	*schn**ei**e* [ɛi], *schn**ii**e* [iː]	7	phon.
*spät* ‘late’	MHG *â*, *æ*, *ô*	*schp**aa**t* [ɒː], schpeät [eə]	12	phon.
*tief* ‘deep’	MHG *ie*, *î*, *û*, *iu*	*t**öi**f*, *t**üü**f*	7	phon.
*trinken* ‘to drink’	MHG *i*, -*nk-*	*tr**eech**e*, *tr**inkch**e*	10	phon.

Table 1 presents the 16 variables selected. The Standard German word, the Middle High German root, example variants, the number of variants, and the type of variable (phonetic, morphological, or lexical) are indicated. The morphological variable ‘upwards’, for example, has 31 different variants, including *embruf* and *uehi*. [16] categorized this as a morphological variable, probably because of its word formation nature. One could also view the 31 variants for ‘upwards’ as different lexical variants. In the present contribution, we adhere to the original *Atlas* categorization.

### 2.2 Mobile implementation

**Fig 2:**
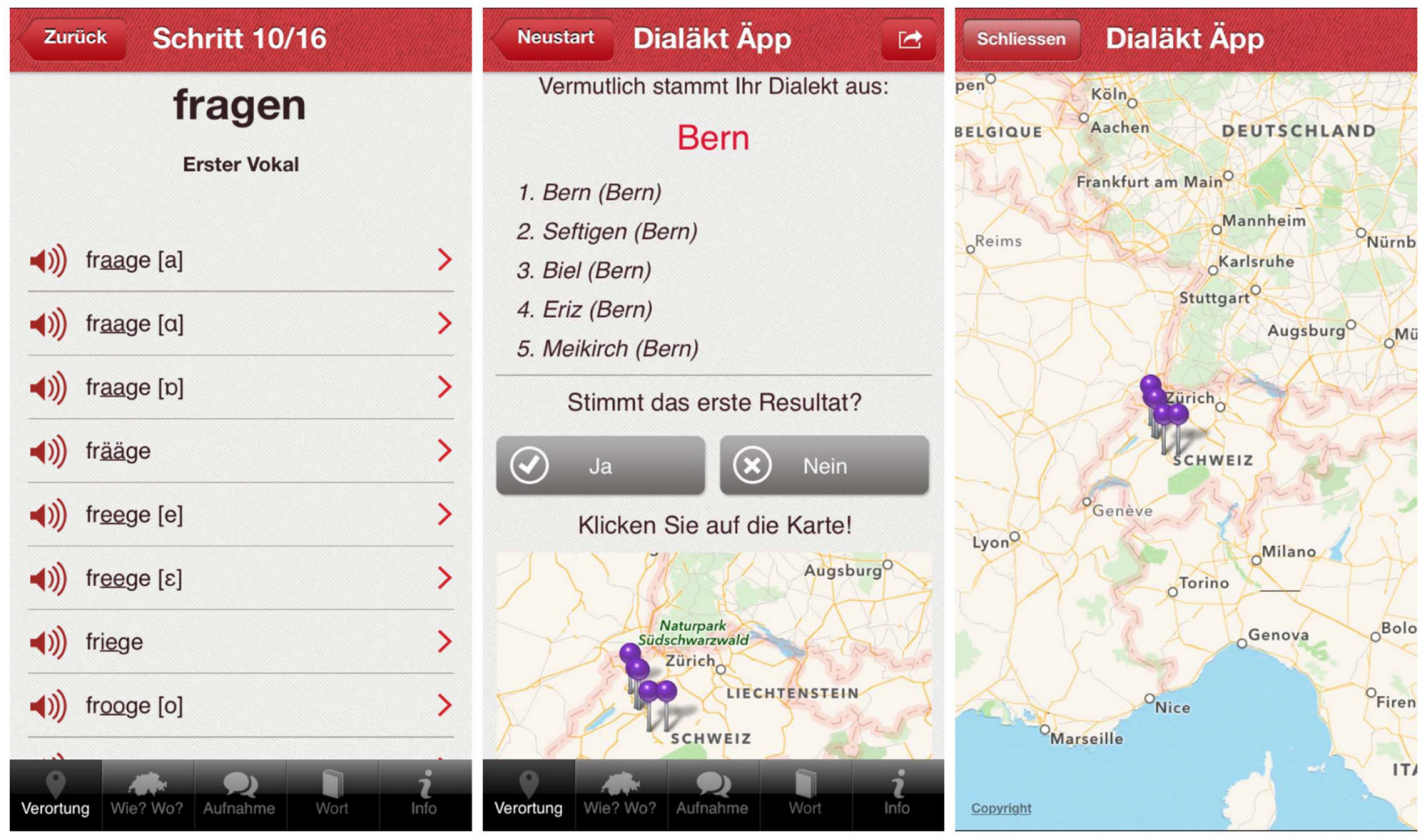
Pronunciation variants for *fragen* ‘to ask’ (left), dialect prediction displayed as a list of five best hits (center) and as pins on a map (right).

DÄ prompts users to select their pronunciation variant from a list for each of the 16 variables by tapping on the smartphone screen. Because Swiss German does not have a standardized writing system, variants are spelled to approximate their pronunciation. When variants cannot be written down because of only minor phonetic differences (e.g. ‘to ask’ (*fraage* [aː], *fraage* [ɑː]; see Fig 2, left), the app further shows phonetic transcriptions. Since users may not be accustomed to these symbols, we included audio recordings for all variants for each of the 16 words. Each variable is presented on a single screen with the Standard German word in its title and dialectal variants listed underneath (Fig 2, left). Once users have indicated which variants of the 16 words they use, the app presents a list of five possible localities, out of a possible 550 adapted from the *Atlas*, that best correspond to their dialect (Fig 2, center) and displays these on a map (Fig 2, right). *Atlas* data from the 16 variables serve as the basis for the dialect prediction algorithm [16]: for each variant of the 16 variables, the *Atlas* contains data on the localities for which the variant is attested. The user input is then compared to this underlying data: a match is observed for a particular locality when the user’s variant for a variable is attested in this locality. Our dialect prediction algorithm calculates scores per locality by aggregating, for each locality, the number of matches. The algorithm then presents the top five localities with the highest scores as best hits to the user [17].

### 2.3 Comparing Atlas with DÄ data

**Fig 3:**
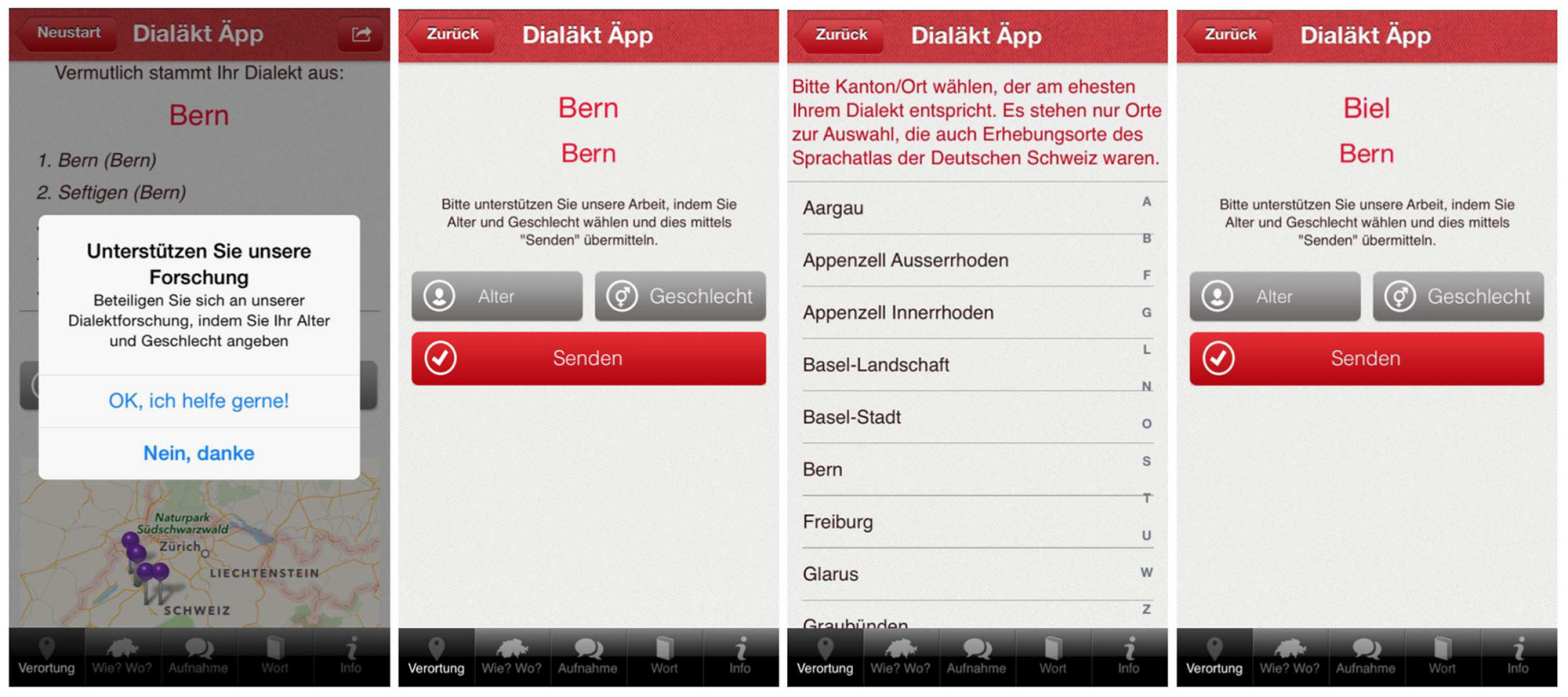
Evaluation of prediction: if satisfied, users are prompted to participate in research (left) by indicating their age and gender (center left). If dissatisfied, users select their locality from a drop-down menu (center right) and then indicate age and gender (right).

In order to conduct analyses of language change on the basis of these data, we need users to provide feedback on the predicted dialect. If they believe the first locality in the result list to be accurate, users are shown a new screen informing them about how to support our research. By clicking on ‘OK, I’d like to help’, users are prompted to indicate age and gender. Having done so, they once again verify their voluntary participation in our study by clicking on ‘send’ to submit their data. In doing so they consent to sending off their data on variant selection, dialectal origin, gender, and age. This is explained on the screen. None of these pieces of information individually or in combination allow for identification of a user in the database. Users also have the opportunity to decline in the first instance, in which case they are shown the results screen again (Fig 3, left and center left). If they feel the result is not accurate, users can specify their dialect by choosing from a list of cantons (administrative regions) and localities (Fig 3, center right), before indicating age and gender. Here too, users have to intentionally click on ‘send’ to submit their data. In doing so they consent to sending off their data on variant selection, dialectal origin, gender, and age. This is again explained on the screen. In both instances—correct or false prediction—the location of a speaker’s actual dialect is elicited. This crowdsourced information can then be compared to the historical data in the *Atlas*. This procedure of collecting and analyzing anonymous user data conforms to the regulations of the Zurich cantonal ethics committee (http://www.kek.zh.ch/internet/gesundheitsdirektion/kek/de/home.html) and the accompanying federal laws on experimentation on humans in Switzerland (http://www.admin.ch/opc/de/classified-compilation/20061313/index.html). For this reason, we did no seek further ethical approval from cantonal or federal institutional bodies.

The localities provided in the drop-down list are identical to those used in the *Atlas*. This allows for direct cross-comparison between *Atlas* data and DÄ data. There are, of course, communities that have merged or changed names in the past 60 years (cf. [30]). The original *Atlas* locality set for German-speaking Switzerland was 560; that of DÄ is 10 localities fewer, i.e. 550.

## Results

In 3.1 we discuss the descriptive statistics of the DÄ corpus, and 3.2 presents the results on the prediction accuracy of DÄ. The rest of the results section is dedicated to an analysis of language change on the basis of the present data (3.3). This includes analyses of variable types (3.3.1), the mapping of specific variables (3.3.2), the effects of the number of variants per variable (3.3.3), and of age on linguistic change (3.3.4). The dataset underlying these findings can be found in the Supporting Information (S1 Dataset).

### 3.1 Descriptive Statistics

#### Number of users

From a total of over 78,000 downloads, 58,923 users indicated their dialect, meaning that either the app predicted their dialect (i.e. canton and locality) correctly and they sent back confirmation, or the app did not provide an accurate prediction and the users self-declared their actual dialect instead. Some people, therefore, received a prediction but decided not to provide feedback, and some downloaded the app but did not use the prediction function.

#### Areal distribution

**Fig 4:**
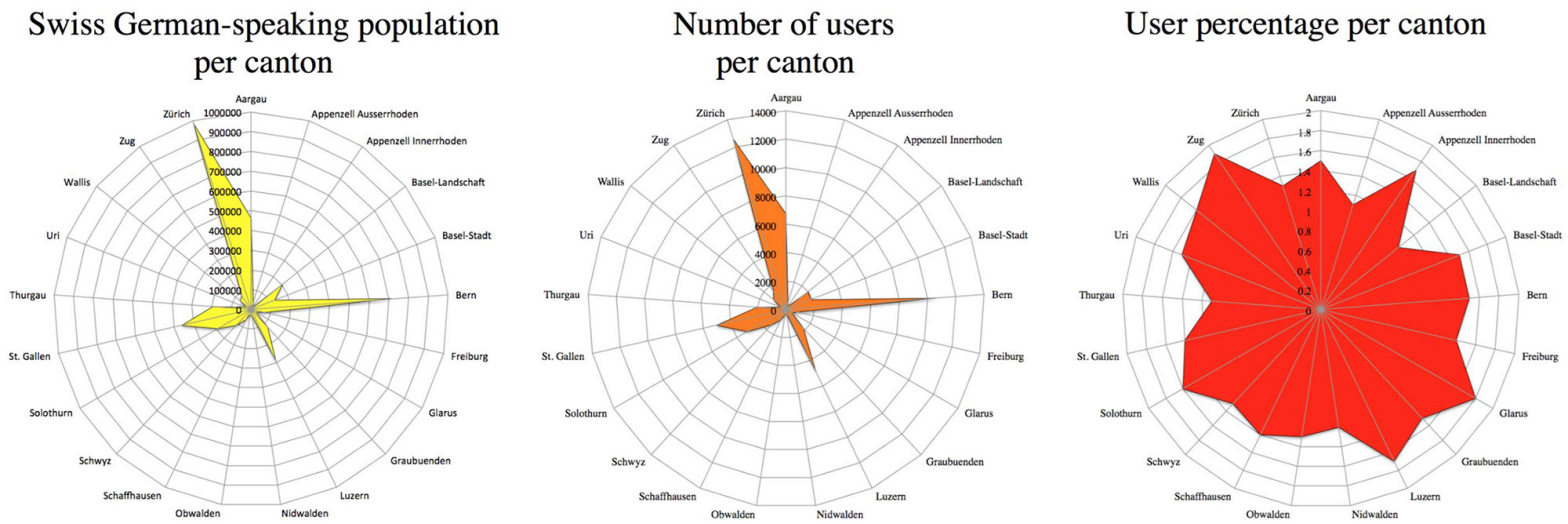
Swiss German-speaking population per canton (left), number of users per canton (center), and user percentage per canton (right).

21% (n = 12,550) of the users were speakers from the canton of Zurich, 18% (n = 10,589) from the canton of Bern, and 11.50% (n = 6,774) from the canton of Aargau. Appenzell Innerrhoden (0.4%, n = 209) provided the lowest number of users. It is not surprising that more than half of all users came from the cantons of Zurich, Bern, and Aargau, given that the majority of Swiss-German speakers live in these three cantons. A closer look at the number of users per city reveals that the city of Zurich alone provided 5.3% of all users (n = 3,119), Bern 4.6% (n = 2,736), Basel 3.1% (n = 1,842), and Luzern 2.8% (n = 1,639). Fig 4 shows the Swiss German-speaking population per canton (left, as indicated by the 2012 Census available from the Swiss Federal Statistics Office (SFSO), Swiss Statistics Website), the number of users per canton (center), and the user percentage per canton (right, i.e. (number of users per canton/Swiss German-speaking population per canton) x 100).

Each radius in Fig 4 represents a canton where German is an official language. The length of the radius stands for the number of Swiss German speakers in that canton (left), the number of DÄ users per canton (center), and the percentage of DÄ users measured by the number of Swiss German speakers in that canton (right). With nearly 1 million people, the canton of Zurich shows the greatest number of Swiss German speakers in Switzerland, followed by Bern (711,000) and Aargau (464,000). The fewest Swiss German speakers are found in Obwalden (28,000) and in Appenzell Innerhoden (12,000). The number of users relative to the number of Swiss German speakers reveals that, on average, DÄ users make up 1.38% of the Swiss German-speaking population per canton (median = 1.45%). The figure ranges from nearly 2% in Zug and 1.8% in Glarus to 1.1% in Thurgau and 1% in Basel-Landschaft.

#### Localities

**Fig 5:**
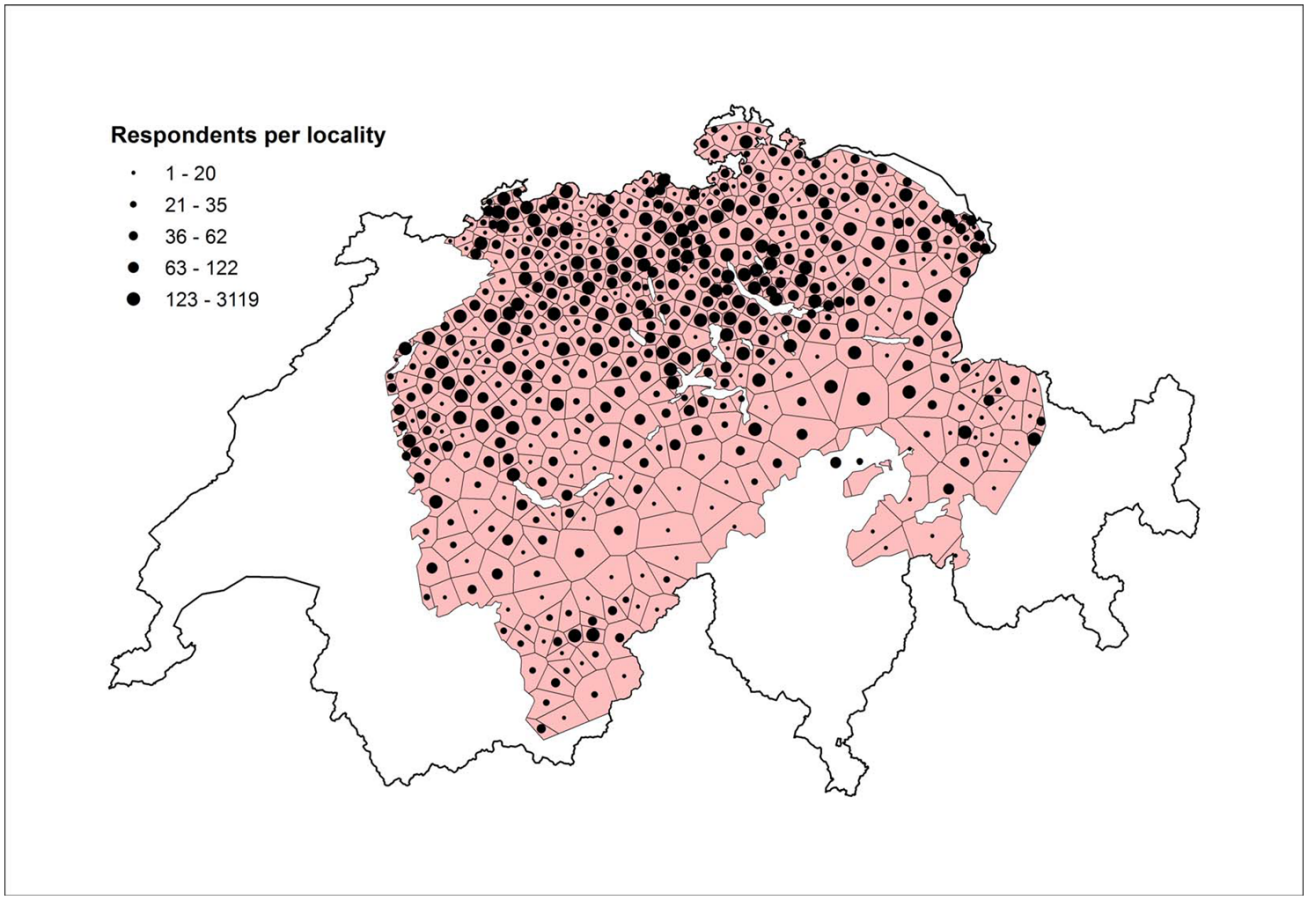
Number of respondents by locality. Each Thiessen polygon represents one locality. The larger the black dot, the more respondents per locality. Polygons are based on Swiss commune centroids derived from generalized commune boundaries available from Swiss Federal Statistics Office (SFSO), Swiss Statistics Website.

The 550 predicted localities are based on those used in the *Atlas* (see 2.3). Descriptive statistics reveal that three places from the *Atlas* were not represented in the DÄ dataset at all: Mutten (GR), Obergoms (VS), and Sternenberg (ZH). For every other locality there was at least one respondent. On average, we observe 107 users per locality (median = 48). Fig 5 shows the number of respondents per locality. We observe that the majority of users are found in the more densely populated Swiss Central Plateau, in the north. The southern, more mountainous localities provided fewer respondents.

#### Gender

Of the users, 42% (n = 24,654) were females and 58% (n = 34,269) were males.

#### Age

On average, users were 31.5 years old with a standard deviation of 15.5 years (median = 27).

### 3.2 Prediction accuracy

30% of users were predicted in the correct locality. When simply considering the cantons and not the localities, the majority of users were predicted in the right canton (65%). The distribution of the number of users that were localized in the right canton, for each canton, was very similar to the distribution of the number of users per canton (χ^2^(324) = 342, p = .236). This entails that the dialects of users from the different cantons were predicted similarly well. When comparing different age groups, the results reveal that prediction accuracy (on a cantonal level) increased with users’ age: the worst predictions were associated with speakers aged 15–20 (59%), 21–25 slightly better (64%), 26–35 even better (66%), and 36–60 (69%) the second best. The oldest speaker group (60+) was predicted the best with a rate of 71%.

### 3.3 Analyses of language change

#### 3.3.1 Variable type

**Fig 6:**
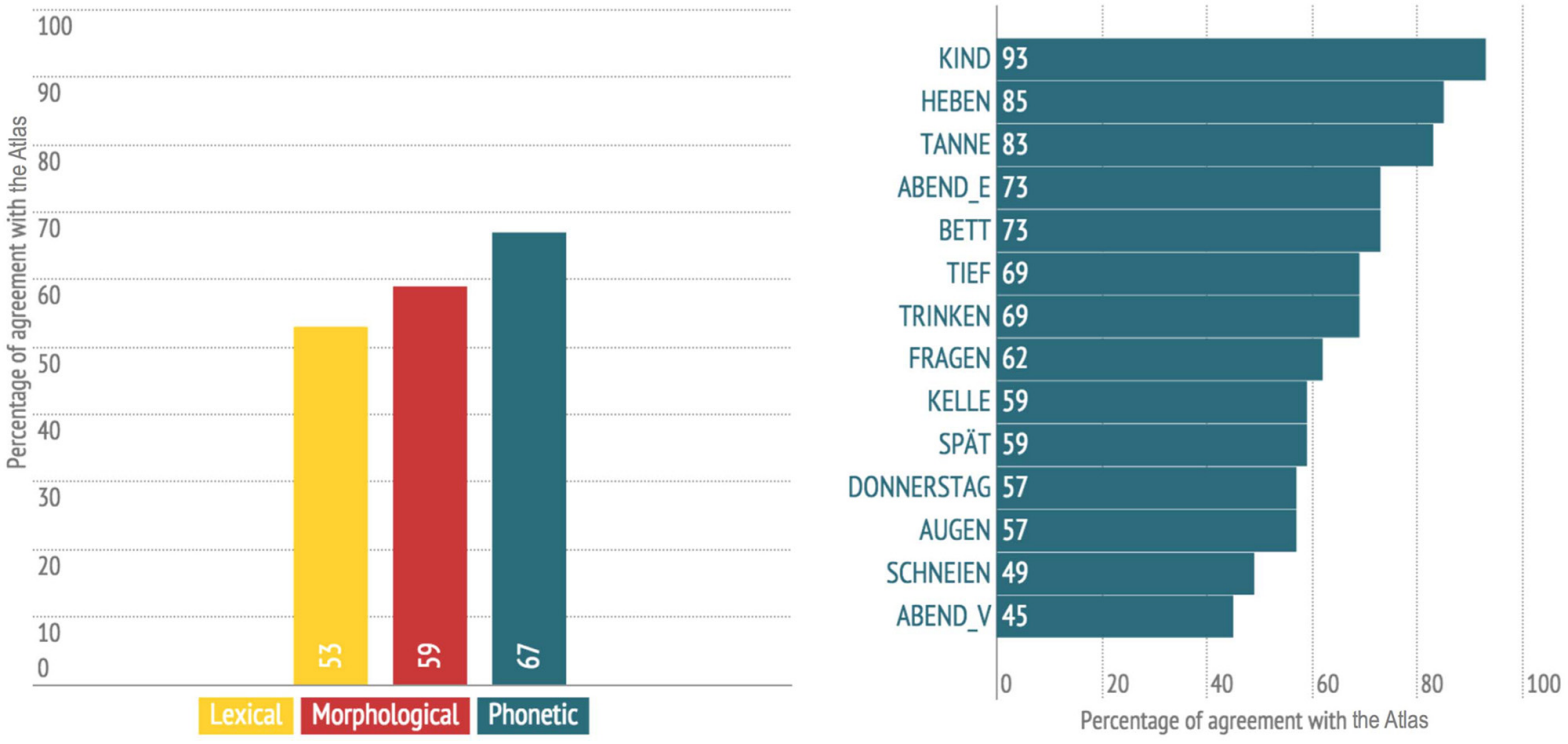
Agreement with *Atlas* by variable type (left) and for each phonetic variable (right).

Three types of variables were used for the prediction of the user’s dialect (see Table 1): phonetic, lexical, and morphological variables. Phonetic variables were disproportionately represented, with 14 out of 16 variables. Only one lexical variable (*Apfelüberrest*, ‘apple core’) and one morphological variable (*hinauf*, ‘upwards’) were used. To analyze language change, we calculated the percentage of agreement with the *Atlas* for each variant. For the variables investigated, the *Atlas* usually shows one variant per variable for each locality. In the cases where the *Atlas* indicates two different variants for one variable in a locality, each of the variants could potentially agree with the users’ variant. If a user’s variant agreed with one of two *Atlas* variants, we counted this as an agreement. In the DÄ corpus, each variant is indexed with the proportion of speakers using that variant per locality. An agreement score of 45% in *Abend* (vowel), for example, means 45% of the users still use the same variant as indicated in the *Atlas*, while 55% do not. Fig 6 shows the percentage of agreement with the *Atlas* for each type of variable (left) and, in greater detail, for each phonetic variable (right).

Phonetic variables show the highest degree of agreement with the *Atlas* (67%) followed by the morphological variable (59%) and the lexical variable (53%). The variable *Kind* ‘child’, for example, shows an agreement score of 93%; for 93% of the users, *Kind* ‘child’ still shows the same variants as documented in their local dialect in the middle of the 20^th^ century. The verb *heben* ‘to lift’ and *Tanne* ‘fir tree’ also reveal high agreement scores with 85% and 83% respectively. Variables such as *Augen* ‘eyes’, *schneien* ‘to snow’, and *Abend* (vowel) ‘evening’, however, have much lower agreement scores. For *Abend* (vowel), for example, less than half of the users indicate the variant that was documented in their locality 70 years ago.

#### 3.3.2 Analyses of specific variables

We now explore three of these variables in greater detail, presenting comprehensive results for *Apfelüberrest* ‘apple core’, the only lexical variable, as well as two phonetic variables: *heben* ‘to lift’ and *Kelle* ‘ladle’. The former phonetic variable exhibits a high agreement score of 85%, while the latter shows a lower score of 59%.

**Fig 7:**
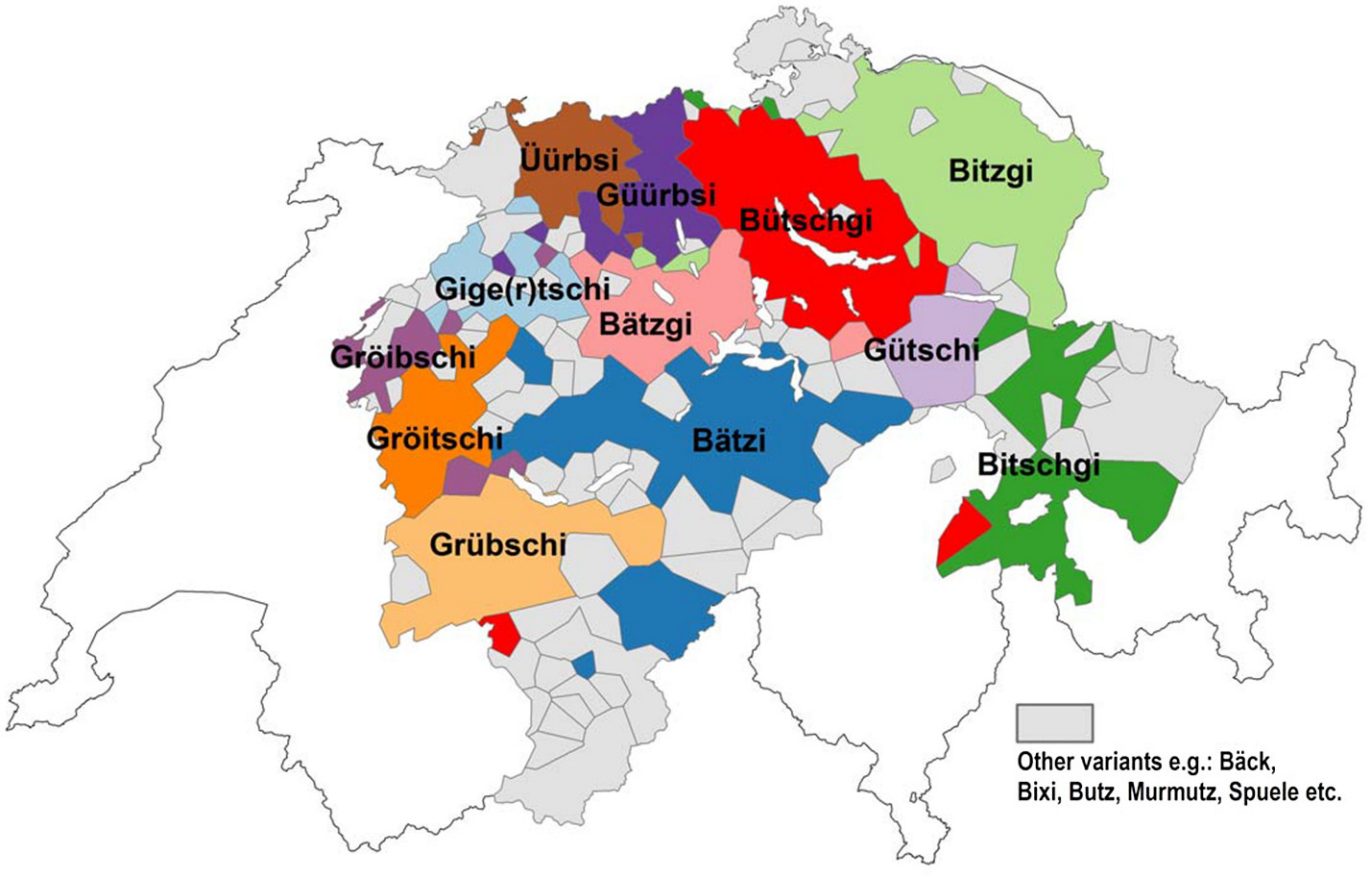
Distribution of variants for *Apfelüberrest* (*Atlas*).

**Fig 8:**
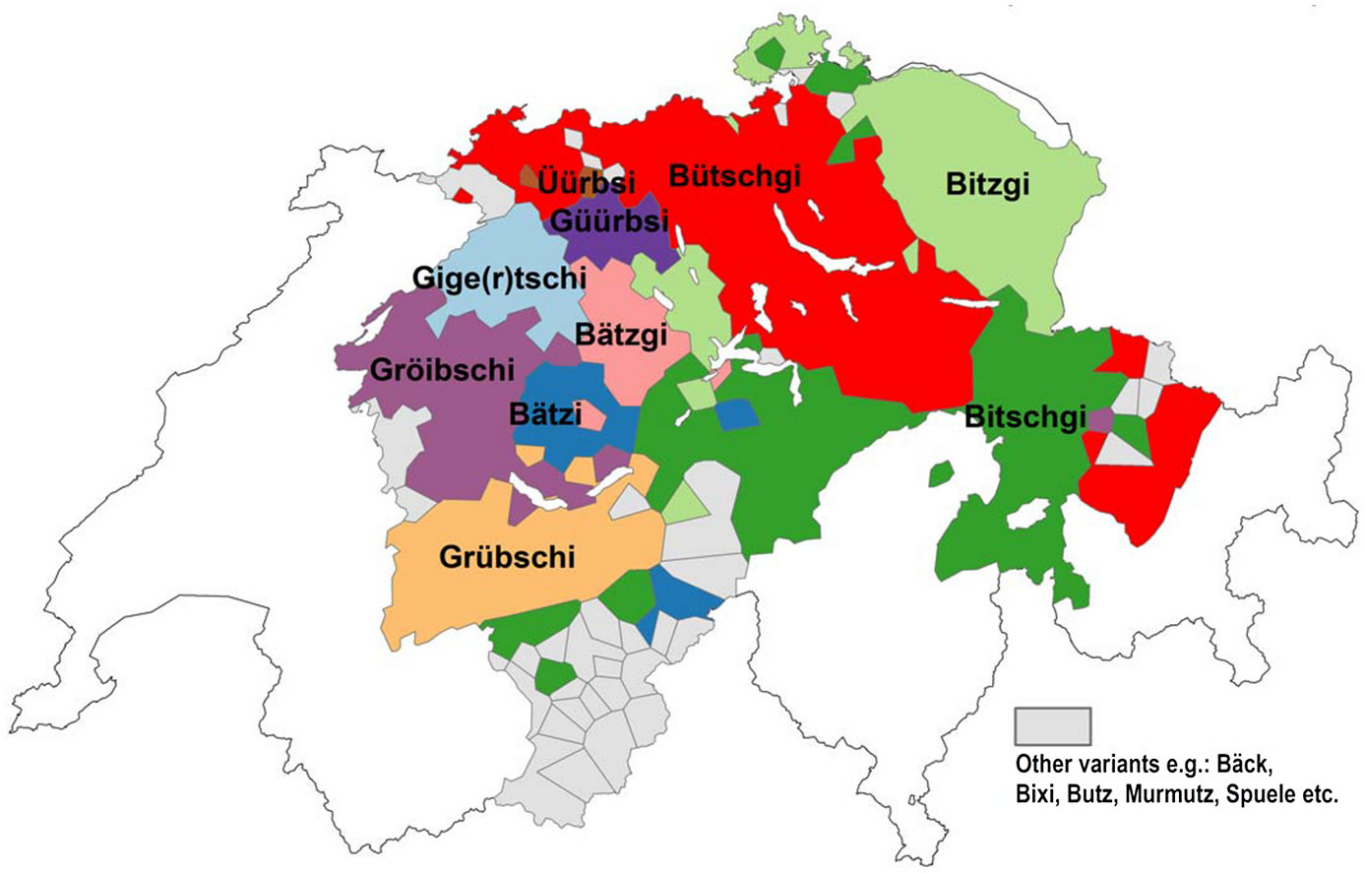
Distribution of variants for *Apfelüberrest* (DÄ).

*Apfelüberrest* has 39 different dialectal variants (see Table 1). An areal representation of the individual variants reveals distinct differences between historical and contemporary data. Figs 7 and 8 show the distribution of *Apfelüberrest* according to the *Atlas* and according to DÄ data as polygon maps. The most dominant variant in each locality, i.e. in each polygon, is depicted.

All the variants that were reported 70 years ago are still in use in the contemporary data, though a number were reported with very low counts (e.g. *Grääni*, n = 24; *Huusini*, n = 36). *Bütschgi* was reported most often (n = 22,587), followed by *Bitzgi* (n = 8,158). The most evident difference between Figs 7 and 8 are the areas colored red, denoting the regional distribution of *Bütschgi*, which has gained ground over the past 70 years diffusing towards the south, west, and northwest. *Bitschgi*, shown in dark green, has also spread extensively, in particular towards central Switzerland. *Gröitschi*, which used to be heard primarily in Western Switzerland, was barely present in the 2014 DÄ data.

**Fig 9:**
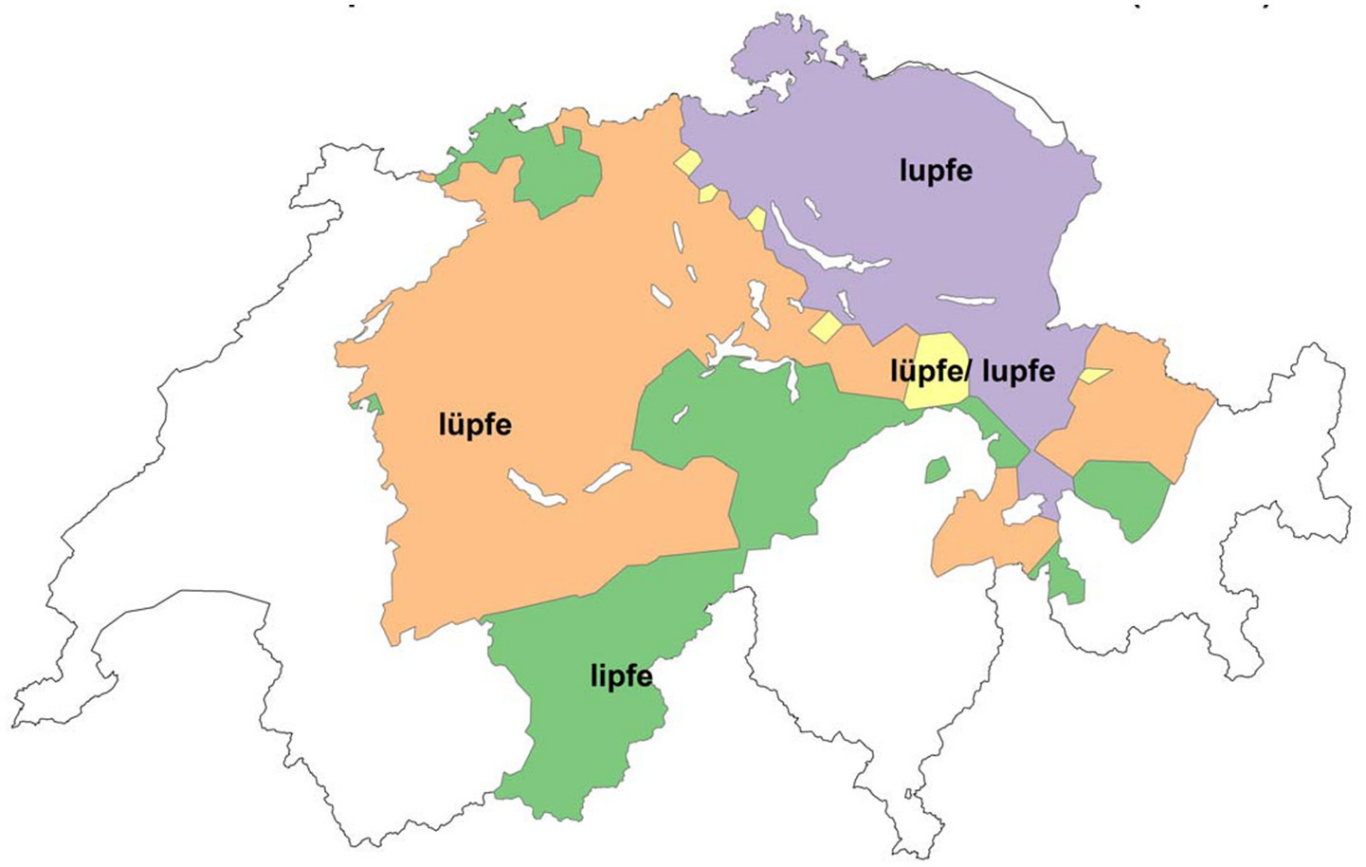
Distribution of variants for *heben* (*Atlas*).

**Fig 10:**
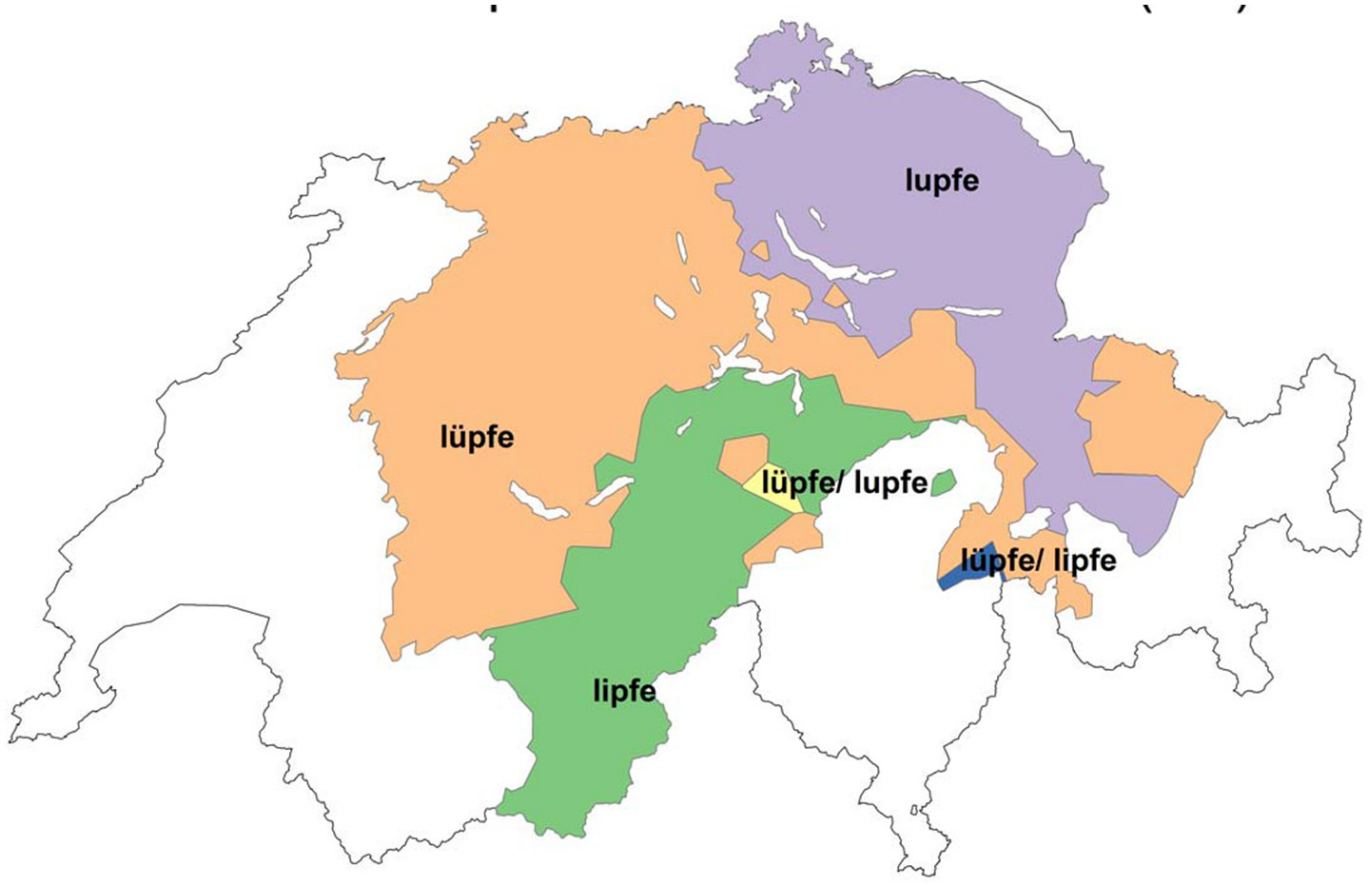
Distribution of variants for *heben* (DÄ).

One variable that exhibits a high agreement score (85%) with the *Atlas* is the phonetic variable *heben* ‘to lift up’. The Swiss German word for Standard German *heben* has three variants: [lupfə], [lypfə], and [lipfə], spelled as <lupfe>, <lüpfe>, and <lipfe>. Figs 9 and 10 show the distribution of *heben* according to *Atlas* data (Fig 9) and DÄ data (Fig 10):

When comparing the two figures, the high amount of agreement becomes evident: in particular, the northeast/southwest isogloss for [lupfə] and [lypfə] seems to be completely stable. One significant difference between the two maps is that the region around Basel, northwestern Switzerland, nowadays prefers the rounded version [lypfə] as opposed to the more traditional and historically attested unrounded variant [lipfə]. Moreover, in south-central Switzerland, regions that traditionally used [lipfə] seem to form an island of [lypfə] in present day data. Note, however, that data from these areas is sparse in the DÄ corpus, with only 3–15 respondents per locality. A notable area in the southeastern canton of Bern shows [lipfə] in our data where the *Atlas* documented [lypfə]. This is based on several localities containing 11–47 respondents.

**Fig 11:**
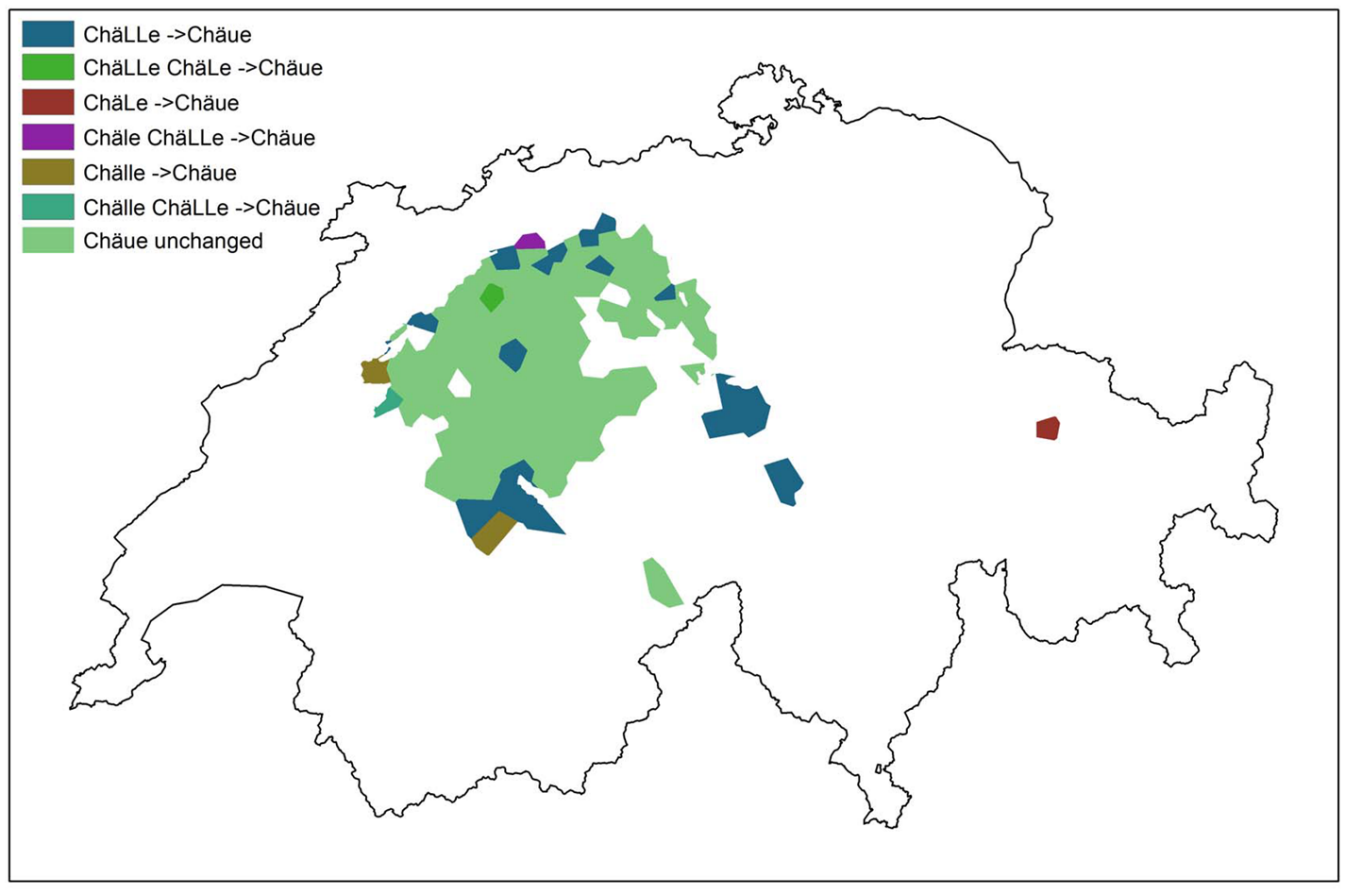
Distribution of vocalized variants in *Kelle*. Light green denotes areas where the *Atlas* had documented vocalization and DÄ shows the same result. Colors other than light green show regions where nowadays DÄ shows vocalization, but the *Atlas* did not.

One phonetic variable that exhibits a low agreement score (59%) with the *Atlas* is *Kelle* ‘ladle’. *Kelle* has five variants, where <ll>, i.e. /l/ can be pronounced as: [l], [lː], [ʊː], [ɬ], [ɬː]. The realization of /l/ as [ʊ], or l-vocalization, has been reported to be diffusing in Swiss German [31, 32]. Fig 11 shows the distribution of this vocalized variant, [ˈxæʊːə], as represented in the contemporary DÄ corpus. Bright green indicates where the *Atlas* indicated /l/-vocalization 70 years ago and where DÄ still indicates /l/-vocalization. Other colors show regions where nowadays DÄ data also shows [ˈxæʊːə], but which used to have a lateral or velarized realization of /l/ 70 years ago (for instance, in the brown spots, *Chälle* was the *Atlas* variant but *Chäue* is now widespread).

In Fig 11, lateral articulations are coded as /xælə/ <Chäle>, velarized /xæɫə/ as <ChäLe> and vocalized /xæuːə/ as <Chäue>. Results reveal an expansion of /l/-vocalization from western Switzerland towards southwestern Switzerland (Bernese Oberland), as well as central Switzerland (towards the southeast), the west, and the northwest. The area showing vocalization in southeastern Switzerland (colored in maroon red), stemming from respondents in Lüen and St. Peter-Pagig, seems to be an outlier. Here, a small number of respondents, two out of three, indicated vocalized variants.

#### 3.3.3 Agreement scores and number of variants per variable

**Fig 12:**
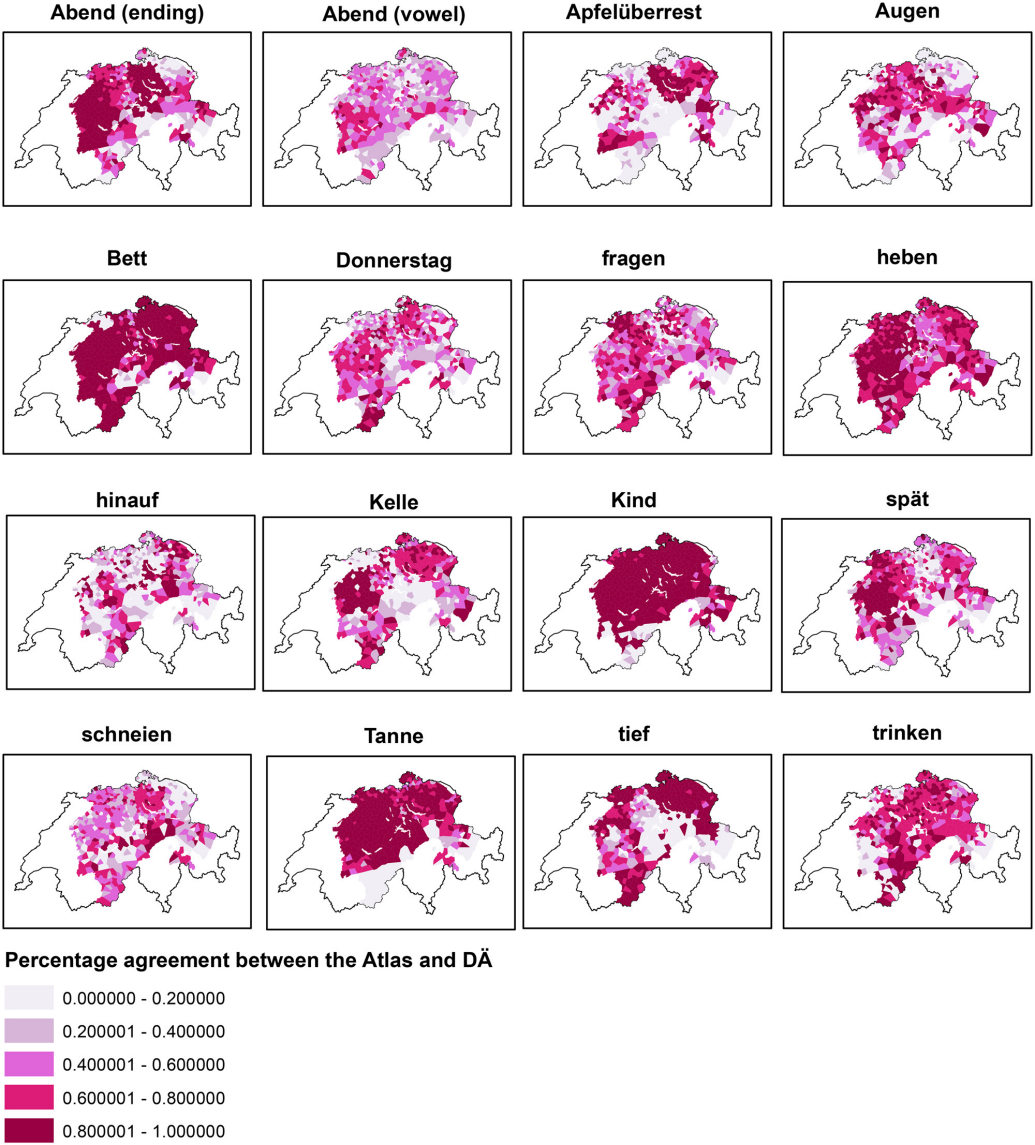
*Atlas* agreement scores by variable. The darker the purple, the higher the agreement with the *Atlas*.

One phenomenon that stands out when considering the agreement scores (Fig 6) is the possibility that variables with a high number of dialectal variants to choose from could inherently have lower agreement scores than those with fewer variants. Fig 12 shows the *Atlas* agreement scores for each variable: the darker the purple, the higher the agreement with the *Atlas*.

**Fig 13:**
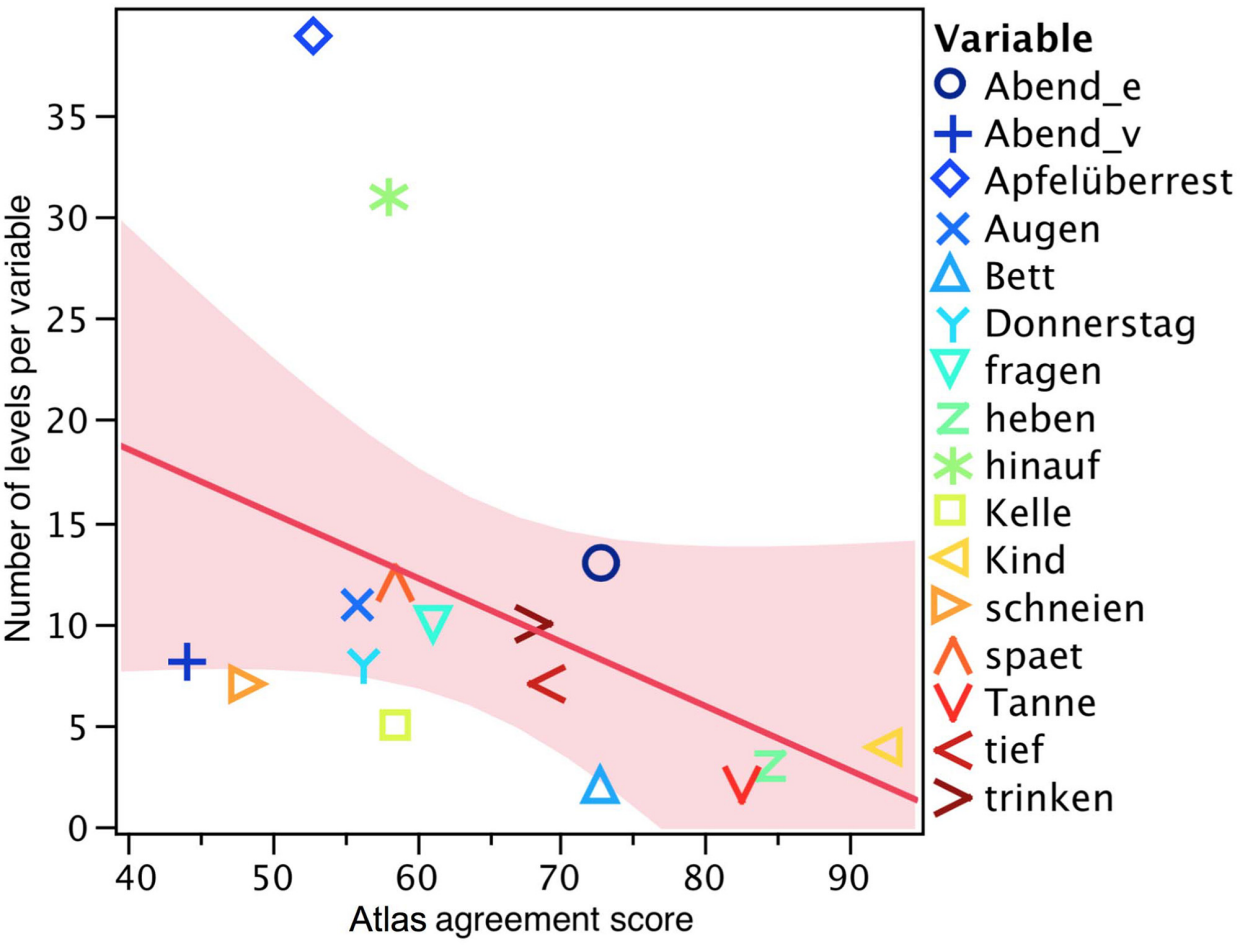
Scatterplot matrix of number of levels per variable as a function of *Atlas* agreement score.

The variables *heben* ‘to lift’ (3 variants) and *Tanne* ‘fir tree’ (2 variants) are examples of this trend, revealing agreement scores of 85% and 83% respectively. In these instances, Fig 12 shows many dark purple areas, i.e. high agreement scores. The opposite trend is visible for *Abend* (vowel) ‘evening’ (8 variants) and *Apfelüberrest* ‘apple core’ (39 variants), with only 45% and 55% agreement scores respectively. This is shown as brighter purple areas in Fig 12. This trend is illustrated in the scatterplot matrix shown in Fig 13.

Fig 13 shows the scatterplot of *Atlas* agreement scores and number of variants per variable. The red line indicates the regression line and the red area denotes the 95% confidence limits. A number of agreement scores scatter perfectly along the regression line (e.g. *trinken* ‘to drink’ and *spät* ‘late’), while others lie somewhat farther away from the regression line (e.g. *hinauf* ‘upwards’, *Abend* (vowel) ‘evening’). We computed a correlation of the number of variants per variable and the *Atlas* agreement scores. The correlation was not significant (Pearson’s correlation: *R*(16) = -0.42, p = .105). A correlation of -0.4 is weak to moderate in magnitude. Despite the statistically non-significant p-value, there seems to be a trend towards variables with more variants having lower agreement scores (see Discussion).

#### 3.3.4 Agreement scores and age

**Fig 14:**
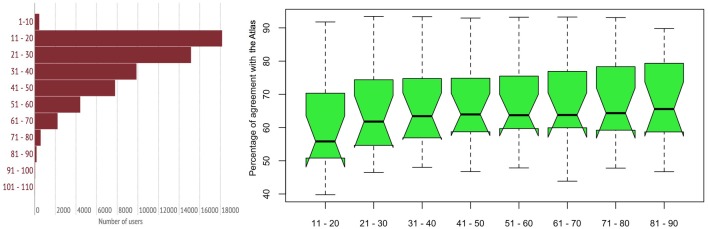
Distribution of number of speakers per age group (left); age group by agreement scores (right). Groups 1–10 as well as 91–100 and 101–110 are not displayed.

We also tested whether there was an effect of age and *Atlas* agreement scores. Speakers were split into equidistant age intervals, ranging from 1 to 110. The distribution of the age groups is shown in Fig 14 (left). By far the largest age group is 11- to 20-year-olds, comprising 31% of all users (n = 18,193). The second largest group is the 21- to 30-year-olds (26%, n = 15,174).

We calculated a linear model to test for an effect of *age* between the speaker groups. Here, the groups of 1–10, 91–100, and 101–110 were excluded. It is plausible to assume that—in these specific cases—many users were not sincere when indicating their age. Also, the top and bottom choices—which are 1–10 and 101–110 –are probably over-represented because they are the easiest to scroll to and to click on within the app. Boxplots of these age groups’ percentage of agreements are shown in Fig 14 (right). There is a tendency for older speakers to have higher agreement scores. The linear model did not provide a significant effect, though.

## Discussion

In this section we touch upon the innovative aspects of our method (4.1) and the app’s public reception (4.2). This is followed by a more thorough discussion of this study’s main results: the app’s prediction accuracy (4.3) and the evidence for language change (4.4). Given that our method does not have a precedent, a large bulk of this discussion is dedicated to methodological considerations (4.5).

### 4.1 The innovative aspect of crowdsourcing language change with an app

The use of crowdsourcing methods to investigate language change is, at present, unusual. Generally, scholars of language variation and change work with very small samples of speakers (often fewer than 50), who have been carefully selected to meet a range of social profile criteria, and do so in one or just a few locations—rarely more (see the discussion, for example, in [33], about the impracticability of large random samples in traditional approaches to sociolinguistic dialectological fieldwork). The search for ‘authenticity’ has long preoccupied dialectologists, and consequently there has been a tendency in the discipline to favor both native and working-class speakers of the local dialect in an attempt to access a location’s ‘true’ vernacular speech (see, for example, [34, 35]). One consequence of such an approach is that while change at a very specific, local level is brought into sharp focus, a general overview at the regional or national level becomes impossible because of gaps in coverage and inconsistency in sampling strategies [36]. However, attempts at securing a representative sample of a given population are beginning to be abandoned, largely due to the expense of securing a truly meaningful sample and the laborious and time-consuming analytical techniques usually applied to collected data [33, 37]. Some notable larger-scale projects that could be seen as crowdsourced include Labov, Ash and Boberg’s phone survey for the *Atlas of North American English* [38], and, especially, Chambers’ *Dialect Topography* of Canadian English, a questionnaire-based survey of regional lexical and pronunciation differences that had, by 2006, been completed by over 6,000 people [39]. In studies of language change, these techniques are, however, the exception rather than the rule.

Crowdsourcing large amounts of dialect data has the potential to complement existing data collection techniques and to provide evidence that traditional methods cannot, with normal resources, hope to gather. As we will see, such techniques offer the opportunity to collect potentially representative samples cheaply and effectively and provide a geographical overview that smaller, more typical samples cannot.

### 4.2 Public reception

DÄ was well received in Switzerland. It peaked as the most downloaded app in the country for a few days in March 2013 and was the most downloaded educational app for several consecutive weeks. The app was extensively covered in daily and weekly newspapers, on Swiss National Radio, and Swiss National Television. This buzz helped promote the app, which led to further recruiting of users, i.e. subjects. App-based experimenting creates its own dynamics. Users were not offered any payment; pure enjoyment and curiosity about Swiss German dialects led them to participate. The app’s popularity is interesting as it is commonly assumed that users expect a gain for the time spent on crowdsourcing tasks [5, 40]. DÄ was not designed as a task, but as playful software to communicate about science; so it seems that the “task” took on a form of enjoyment for the users—which may have been even more effective at recruiting participants than a monetary incentive.

### 4.3 Prediction accuracy

The evaluation of prediction accuracy was somewhat sobering: the narrowly-defined first-hit accuracy on the locality level was relatively low at 30%. Accuracy at a broader cantonal level—testing if the first hit was in the right canton—was comparatively high at 65%. An abstraction to the cantonal level is justified insofar as dialect regions are not always clearly delineated in German-speaking Switzerland (cf. [41])–a rule of thumb holds that the dialects are named according to the canton in which they are spoken. This recognition rate is remarkable, given that there are 19 German-speaking cantons, making the chance level 5.3% (= 1/19). This type of analysis is justified since DÄ’s prediction algorithm showed a similar performance for every cantonal dialect (cf. 3.2). This semi-automatic dialect recognition performs better than human dialect recognition: for Swiss German, [42, 43] showed that naïve listeners can accurately recognize another speaker’s dialect with a recognition rate of 86% and 74%, in a four and eight alternative forced choice task, respectively. That is 86% when chance level is 25% (= 1/4) and 74% when chance level is 12.5% (= 1/8). In other languages, however, dialect recognition tends to be more difficult: [44] report human identification rates of 30–50% for American and British English dialects; [45] report similar recognition rates for German dialects. The tendency for younger speakers to have poorer DÄ prediction rates than older speakers is intuitively sound [27]: The older speakers can be expected to be closer, in their speech, to the *Atlas* informants, on which DÄ’s prediction algorithm is based. [25] found older speakers to have higher *Atlas* agreement scores in her sample of 5,500 speakers. For Swiss German, studies have reported that younger speakers are more linguistically flexible, i.e. younger speakers tend to use more geographically diverse features than older speakers [26, 46]. In the present study, too, there was a tendency for older speakers to have higher *Atlas* agreement scores (Fig 14).

### 4.4 Language change

Mismatches between the *Atlas* data and self-reported data from the app suggests linguistic change in progress, i.e. that a dialect has changed between the *Atlas* and now. The reported results therefore point to significant language change over the past 70 years, whether on a phonetic, lexical, or morphological level. We can speculate as to the causes of this change. Greater speaker mobility increases the range of variants that speakers may come into contact with, favoring the transmission chances of more frequently used and geographically more widespread variants over rarer and more isolated forms [47]. Swiss German speakers’ increased tendency to send text messages and contribute to social media *in dialect* may also increase speakers’ contact with a diverse range of linguistic variants [25]. Some have argued [48], though not without controversy (see [49] and the series of discussant papers thereafter), that mass media may contribute to language change. Our results further reveal a possible scaling of variables in language change: phonetic variables seem to be less affected than lexical ones, a finding also attested elsewhere [50]. The lexical variable *Apfelüberrest* (‘apple core’) showed an agreement with the *Atlas* of 53%; that is, nearly half of our respondents chose a different variant from the one indicated in the *Atlas*. Phonetic variables, on the other hand, seem to be more stable, with 67% of speakers still adhering to forms documented for that location in the *Atlas*. [26] has previously shown that for Swiss German, phonetic variables seem to be more resilient against language change. The detailed analyses of *Apfelüberrest* ‘apple core’ and *heben* (‘to lift’) exemplify this. *Bütschgi*, one variant of *Apfelüberrest*, is clearly gaining ground and spreading, while *heben* is an example of a phonetic variable that has remained relatively geographically stable over the past 70 years.

These findings have to be interpreted cautiously, however. There were only one lexical and one morphological variable included in the sample, while phonetic variables make up 14 of the 16 investigated. Besides, results reveal a slight trend showing that the more variants a variable has, the lower the *Atlas* agreement scores. This may, on the one hand, have to do with the fact that users were overwhelmed with the number of variants to choose from and had difficulties telling apart the fine-grained phonetic differences, but, on the other, may simply be a reflection of a trend, in the context of increasing mobility, to level away extreme dialect diversity [36, 51]. In particular, it is the variables *Abend* (vowel) ‘evening’ (eight variants), *schneien* ‘to snow’ (seven variants), and *Augen* ‘eyes’ (eleven variants) that exhibit the lowest *Atlas* agreement scores (see Fig 6).

### 4.5 Methodological considerations

There are a number of methodological issues that warrant further discussion. The results of this study need to be interpreted against the backdrop of these limitations:

- The methods used to collect data for the *Atlas* and the methods used for creating the present app-based corpus are different in a number of respects (4.5.1).
- The user’s self-declared dialect, which serves as a basis for analyses of language change here, could be viewed as somewhat problematic (4.5.2).
- There are other methodological concerns with using crowdsourced data that deserve mention (see 4.5.3).

#### 4.5.1 *Atlas* vs. App crowdsourcing

The *Atlas* and DÄ are corpora are based on different methods:

**Data elicitation**: The *Atlas* data were collected using a direct method. Researchers went into the field, conducted interviews, and had subjects fill out questionnaires. The *Atlas* was generated based on the answers provided. DÄ data were collected indirectly, with no researcher present. There is, therefore, much less control over how the data were elicited in the indirect method. One could argue, however, that one advantage of the app-based corpus is that every user receives essentially the same stimulus to respond to, whereas in the *Atlas* (as in other similar dialect atlas projects with multiple fieldworkers), it is possible that different data collectors administered the task slightly differently—and transcribed the speakers’ variants slightly differently. Reports of regional dialect ‘differences’ that can be accounted for by different field and transcription techniques being applied by different fieldworkers are not at all uncommon in the dialectological literature (e.g. [52, 53]).

**General criteria of respondents**: For the creation of the *Atlas*, as was typical of dialectology at the time [54], older speakers who had lived in the respective locality for a long time were typically selected. With the app, a broader, more representative sample of the speakers of Swiss German was targeted. The users recruited in the DÄ corpus come from a much wider range of Swiss German linguistic backgrounds, educational levels, and mobility habits compared to the subjects recruited for the *Atlas*. Furthermore, because the researcher using the app technique has no real control over the sample using the app, we can exclude any potential sampling bias that might be driven by an investigator’s search for the most ‘authentic’ speakers (see 4.1).

**Speaker age**: While *Atlas* subjects were mostly from the older generation, most often between 51 and 80 years of age [55], the average age of the speakers in the DÄ corpus was 32, with a median of 27, indicative of the well-known digital divide [56]. This difference in age groups in the two corpora affects the interpretation of the temporal difference between the two corpora [25]. The *Atlas* is said to reflect the linguistic situation of the first decades of the 20^th^ century [55]. The high percentage of younger participants in the DÄ corpus entails that the DÄ corpus reflects language use in the early 21^st^ century.

**Number of speakers**: The number of speakers in the two corpora is probably what is most different. While the *Atlas* usually had two speakers per locality, DÄ on average has 107 (median = 48) speakers per locality. Because of this large number of respondents per locality, the DÄ corpus may paint a more objective picture of the Swiss German speaking population. The 560 *Atlas* subjects corresponded to roughly 0.019% of the Swiss German-speaking population in 1950. The 58,923 users in the DÄ corpus correspond to 1.1% of the present-day Swiss German-speaking population.

#### 4.5.2 Self-declared dialect

Users were asked to provide feedback on whether or not the prediction was correct and, if not, to indicate their real dialect location. We performed the comparative analyses based on this self-declared dialect, since we are forced to assume that users have an understanding both of their linguistic origins and of their own linguistic usage. However, it is possible that users imitated a ‘model’ dialect in their responses, perhaps due to its prestige, which would cause them to be more homogeneous than is really the case [5]. Alternatively, they may well have nostalgically claimed traditional variants from their communities that they themselves no longer use. [57] robustly demonstrates that English speakers have relatively poor intuitions about some aspects of their own non-standard dialect use, though given that the status of dialects in Switzerland is quite unlike the situation in the Anglophone world, we cannot necessarily assume that Swiss German speakers’ intuitions are equally faulty. In fact, the research discussed in 4.3 may support the latter view: Swiss German listeners seem to differ from Anglophone listeners in their perception of dialects, as shown by high dialect recognition performances of the former. Their awareness regarding dialectal variation is thus likely to be higher.

#### 4.5.3 Further limitations of crowdsourced data

There are some pitfalls to crowdsourced dialect data, but also clear benefits. The caveats listed here should be kept in mind when interpreting the results of the present study.

**Perception in crowdsourcing**: When users selected their dialectal variants, they were given the opportunity to listen to recordings (see section 2.2). This should help guide their decision process, given that some dialectal variants feature only small phonetic differences (e.g. vowel quality or consonant quantity). Essentially, users took part in a speech perception test. Such testing in an app environment is not entirely unproblematic, as it entails low contextual control over participants’ physical and social environments. Listeners may do the task at home or at work, in rooms with different amounts of environmental noise, and with or without uncalibrated headphones. Also, subjects are distracted very easily [9]: they may have other applications running on their phones or possibly receive e-mail alerts or instant-messages. This type of experiment contrasts with listening tests conducted in laboratories, typically sound-treated rooms with state-of-the-art equipment, where stimuli are delivered undistorted to the listeners. One upside of these ‘unfavorable’ effects when testing with the app environment is, however, that all these factors increase the validity of the results: the data represent the type of hearing performance achieved in day-to-day life. Consequently, these findings are likely to generalize better to a greater range of real-world situations (e.g. [58]).

Moreover, listeners are known to vary in their perceptual performance: such variation is largely due to differences in exposure to dialects, metalinguistic awareness, age, hearing, and for some tasks, personality and educational factors [59]. When a researcher conducts a perception test, it is common practice to seek a homogeneous group of participants meeting well-defined selection criteria. Criteria that are normally controlled for include gender, age, educational level, language history and normality of hearing. Controlling for hearing, for example, is critical because many people with mild or even moderate hearing loss are unaware of their deficit [59]. Upholding such criteria is difficult with a design such as the one used in DÄ.

**Multiple submissions**: In laboratory research, subjects typically only submit their data once, while app- or web-based research allows for multiple submissions [60]. There are different scenarios that may lead to multiple submissions: (a) the same person uses the same app to participate repeatedly or (b) the same person uses the app on different smartphones to participate repeatedly. [61] suggests, however, that the rate of repeated participations—below 3% in most studies—does not seem to be a significant threat to the trustworthiness of web- and app-based research.

**Sampling bias**: In the current study, only people who have access to an iPhone were able to contribute information on their dialect. The iOS platform was chosen because, at the time, it was the most widely used smartphone platform in Switzerland. By using Android or other platforms, of course, potentially different social substrata could have been reached. Yet even if Android users were present, there is no guarantee that any particular method of recruitment would yield a sample representative of some particular population.

**Response bias**: The input interface may influence how a participant will respond. The dialect variants were presented in a list from top to bottom; in some instances, the user must scroll through the choices to select an answer [60]. It is possible that answers shown on top were more likely to be clicked.

**Experimenter bias**: With the crowdsourcing method, we do not know if users read the instructions given to them. In laboratory experiments, the researcher has the opportunity to explain the procedures and materials to the participants. The chances that the participant understands the instructions are greater, as the researcher can verify and interact with the participant. In an app environment this interaction is difficult to achieve [61].

**Trustworthiness**: The trustworthiness of participants of web- or app-based studies is an oft-cited problem [62]. How can we be sure that the users are providing meaningful responses? Proponents of app-based experimentation note, though, that this question applies to all behavioral testing whether app-based or laboratory-based [59].

**Connectivity**: Smartphones feature different Internet connection modalities that can cause erratic connectivity (such as Wi-Fi or 2G/3G/4G). As a consequence, a one-to-one mapping of devices to servers cannot be guaranteed. Incomplete Internet coverage often means some data is likely to be lost [8]. In the context of DÄ this means that some data sets may not have been transferred due to poor connections.

There are also clear benefits to crowdsourced dialect data, such as low costs for subject recruitment [63]. Conducting the current research using the same methods as applied in the *Atlas* would have been extremely expensive. The development of the app constituted a fixed investment of time and money, which resulted in good value compared to traditional laboratory experiments considering the number of participants recruited. Moreover, apps offer high convenience in terms of audio and video playback via screen displays. Dialect samples for evaluation can be played and recorded directly on the device. iPhones are easy to use and nearly identical for every dialect speaker. Unlike web-based tasks, stimuli can be loaded natively on the phone, which means that no buffering is required; for perception studies—as is the case with DÄ –this means that variables such as onset times and vowel durations can be controlled with millisecond precision [59]. Unlike web-based tasks, smartphones and tablets tend to focus the attention more on one task at a time than is the case with regular computers [64].

## Quality Control and Validating the App Results

Studies that use a crowdsourcing methodology need to pay particular attention to quality control in their data. There are a number of protocols that can be applied to eliminate noise and filter bad judgments. For example: do average user results compare similarly to how experts, i.e. linguists or phoneticians, would respond [5]? Moreover, the data of one user can be compared to that of another user. An early-stage mechanism for improving the quality of data is the calculation of regional averages, for example, or eliminating outliers [65].

**Fig 15:**
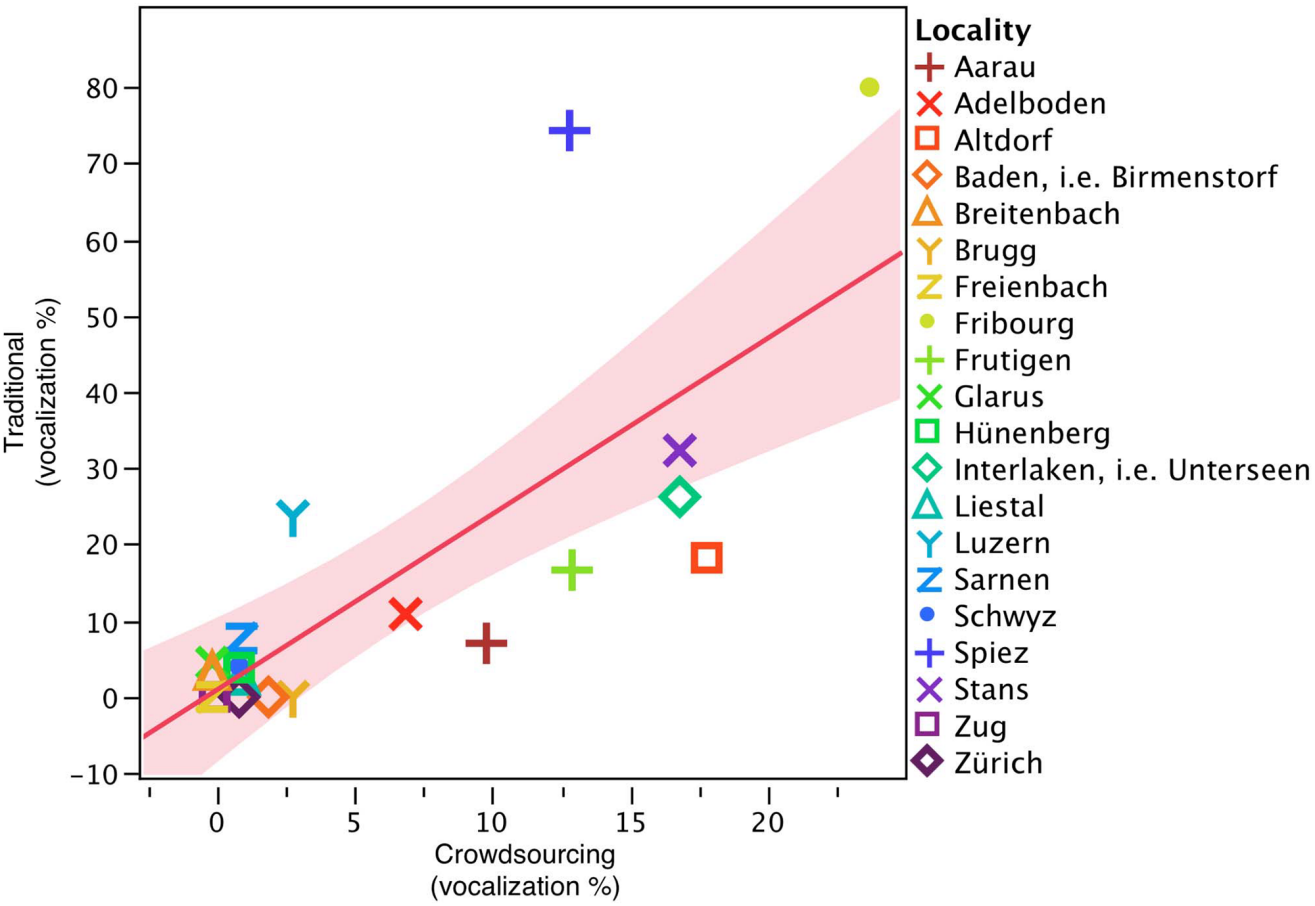
Scatterplot matrix of the degree of vocalization as captured by the traditional method as a function of the degree of vocalization as measured by crowdsourcing.

In the current study, most of these methods are not applicable, however. Outliers can be excluded and means can be calculated. But it is hardly telling to compare one user set to that of another. Experts, too, cannot really help with the validation process, given that each speaker’s linguistic biography is different. Applying various other dialectological methods, however, can validate or falsify results obtained with crowdsourcing [65]. In order to scrutinize the validity of the DÄ corpus, therefore, we decided to examine one particular linguistic variable that has received recent and thorough geolinguistic investigation in Swiss German—the spread of /l/ vocalization, as described in 3.3.2. By means of a well-established dialectological method—a rapid anonymous survey–[32] found a highly similar diffusion of /l/-vocalization in Swiss German to the one presented here. [32] examined 35 native dialect speakers on average (SD = 9) in each of 20 localities (i.e., nearly 700 subjects). They reported diffusion of this typically Bernese German phenomenon towards the west, south, and southeast. That the two methods provide very similar results is illustrated in the scatterplot matrix shown in Fig 15.

Fig 15 shows a scatterplot of the degree of vocalization as captured by the crowdsourcing method (x-axis) and the degree of vocalization as captured by the traditional method (y-axis). Degree of vocalization means the percentage of vocalized /l/ tokens found in these localities. In Adelboden (red ‘x’ symbol in Fig 15), for example, the traditional method documented that 11% of speakers vocalized; in the crowdsourcing method, 7% of the speakers reported vocalization. Not all localities from the [32] study are represented in the *Atlas* (Baden and Interlaken), which is why the closest *Atlas* neighbor was taken as a reference point; for Baden this was Birmenstorf, and for Interlaken this was Unterseen. The red line indicates the regression line, with the red area denoting the 95% confidence limits. A number of agreement scores scatter perfectly along the regression line (e.g. Hünenberg (green square symbol), Freienbach (yellow ‘z’ symbol), and Zürich (bold print purple square)). These localities all indicate a low degree of vocalization in both methods. Other localities lie somewhat farther away from the regression line. Spiez (blue ‘+’ symbol), for example, shows a high degree of vocalization as captured by the traditional method, but a much lower degree in the crowdsourcing framework. Fribourg city (green dot symbol), too, shows a high degree of vocalization in the traditional method, but much less so in DÄ. We computed a correlation of the degree of vocalization as captured with the traditional method and the degree of vocalization as captured with the crowdsourcing method. The correlation was significant (Pearson’s correlation *R*(16) = -.77, p <.0001*), with a strong linear relation between the two variables. There is a clear trend that the degree of vocalization captured by the two methods is related. Given this evidence, it seems that the applied crowdsourcing method provides a valid and promising tool for documenting language change. This is particularly interesting given that /l/-vocalization is a phonetic variable, which for elicitation faces all the many limitations mentioned earlier for crowdsourcing perception data (see 4.5.3).

## Conclusion

DÄ capitalizes on the Swiss public interest in dialectology by providing functionality that allows users to localize their own Swiss German dialect on the basis of a few words. We built a model with a set of maximally predictive words that were chosen from the historic *Linguistic Atlas of German-speaking Switzerland*, which documents the language situation of the mid-20^th^ century. These words highlight the differences between dialect areas. Given that we elicited the user’s dialect with the app, we were able to compare old *Atlas* dialect data to data from 2013/2014, allowing us to locate and document language change. We report that changes have taken place on all investigated linguistic levels: phonetic, lexical, and morphological. Results further show a trend that phonetic variables seem less vulnerable to change, while lexical and morphological variables diverge from the *Atlas* findings to a greater degree. We report overlap in results of this crowdsourcing method with more traditional dialectological approaches, thus underlying the validity of using these new methods for studying language change. The use of smartphones for scientific experimentation potentially heralds a new era in linguistics. DÄ architecture has recently been applied on different languages, including American English [66] and Austrian and German dialects of German [67]. Apps for British English, Japanese, and French dialects are currently in development.

## Supporting Information

S1 Dataset The minimal dataset underlying the findings of the present study. (ZIP)

## References

[1] Merriam-Webster. ‘crowdsourcing’. Merriam-Webster.com; 2014. Available: http://www.merriam-webster.com. Accessed 2015 Apr 20.

[2] Sauer JR, Schwartz S, Hoover B. 1996 The Christmas bird count home page; Version 95.1. Patuxent Wildlife Research Center, Laurel, MD.

[3] Wenker G. Sprach-Atlas von Nord- und Mitteldeutschland. Strassbourg; Trübner; 1881.

[4] Wrede F, Martin B. Deutscher Sprachatlas Auf Grund des don Georg Wenker begründeten Sprachatlas des Deutschen Reichs und mit Einschluss Von Luxemburg, Der deutschen Sprachteile der Tschechoslowakei, Oesterreich, der Sprachinsel Gottschee, Liechtensteins. Marburg: Lahn; 1956.

[5] Eskenazi M. The basics In: Eskenazi M, Levow GA, Meng H, Parent G, Suendermann D, editors. Crowdsourcing for speech processing: Applications to data collection, transcription and assessment. Hoboken, NJ: John Wiley & Sons; 2013 pp. 8–36.

[6] Amazon Mechanical Turk; 2005–2015. Available: https://www.mturk.com. Accessed 2015 Nov 6.

[7] Callison-Burch C, Dredze M. Creating speech and language data with Amazon's Mechanical Turk In: Association for Computational Linguistics, editor. Proceedings of the NAACL HLT Workshop on Creating Speech and Language Data with Amazon's Mechanical Turk 2010, Los Angeles 2010 pp. 1–12.

[8] Brown HR, Zeidman P, Smittenaar P, Adams RA, McNab F, Rutledge RB, et al Crowdsourcing for cognitive science—The utility of smartphones. PloS one. 2014;9: e100662 10.1371/journal.pone.0100662 25025865 PMC4099129

[9] Miller G. The smartphone psychology manifesto. Perspectives on Psychological Science. 2012;7: 221–237. 10.1177/1745691612441215 26168460

[10] Schlippe T, Ochs S, Schultz T. Web-based tools and methods for rapid pronunciation dictionary creation. Speech Communication. 2014;56, 101–118.

[11] de Vries N, Davel MH, Badenhorst J, Basson WD, de Wet F, Barnard E, et al A smartphone-based ASR data collection tool for under-resourced languages. Speech Communication. 2014;56: 119–131.

[12] Iwaidja Inyman Team. Ma! Iwaidja; 2012. Available: https://itunes.apple.com/au/app/ma-iwaidja/id557824618?mt=8. Accessed 2014 Feb 25.

[13] Madnani N, Tetreault J, Chodorow M, Rozovskaya A. They can help: Using crowdsourcing to improve the evaluation of grammatical error detection systems Proceedings of the 49th Annual Meeting of the Association for Computational Linguistics, 2011, Portland, OR 2011: 508–513.

[14] Gonçalves B, Sánchez D. Crowdsourcing dialect characterization through Twitter. PloS one. 2014;9: e112074 10.1371/journal.pone.0112074 25409174 PMC4237322

[15] Leemann A, Kolly MJ. Dialäkt Äpp; 2013. Available: https://itunes.apple.com/ch/app/dialakt-app/id606559705?mt=8. Accessed 2014 Feb 25.

[16] Atlas = Sprachatlas der deutschen Schweiz. Bern (I–VI)/Basel: Francke (VII–VIII); 1962–2003.

[17] Kolly MJ, Leemann A. Dialäkt Äpp: Communicating dialectology to the public—crowdsourcing dialects from the public In: Leemann A, Kolly MJ, Dellwo V, and Schmid S, editors. Trends in Phonetics in German-speaking Europe. Bern/Frankfurt: Peter Lang; 2015 pp. 271–285.

[18] Goldman JP, Leemann A, Kolly MJ, Hove I, Almajai I, Dellwo V, et al A crowdsourcing smartphone application for Swiss German: Putting language documentation in the hands of the users In: Proceedings of LREC 2014, Reykjavik; 2014.

[19] Kolly MJ, Leemann A, Dellwo V, Goldman JP, Hove I, Almajai I. Voice Äpp. A smartphone application for crowdsourcing Swiss German dialect data In: Proceedings of Digital Humanities 2014, Lausanne; 2014 pp. 231–233.

[20] Katz J, Andrews W. How y’all, youse and you guys talk. New York Times Online; 20 Dec 2013. http://www.nytimes.com/interactive/2013/12/20/sunday-review/dialect-quiz-map.html?r=0. Accessed 2013 Dec 21.

[21] Vaux B, Golder S. The Harvard dialect survey. Cambridge, MA: Harvard University Linguistics Department; 2003.

[22] Katz J. Beyond soda, pop, or coke; 2013. Available: http://www4.ncsu.edu/∼jakatz2/files/dialectposter.png. Accessed 2015 Nov 6.

[23] New York Times web analytics group. Available: http://www.nytco.com/wp-content/uploads/2013-Most-Visited-1.png. Accessed 2015 Nov 6.

[24] Glaser E. Der Wortschatz des Schweizerdeutschen; 2008. Available: http://www.ds.uzh.ch/Forschung/Projekte/Schweizer_Dialekte/index.php. Accessed 2014 Feb 25.

[25] Juska-Bacher B. Wortgeographischer Wandel im Schweizerdeutschen. Sommersprossen, Küchenzwiebel und Schmetterling 70 Jahre nach dem SDS. Linguistik online. 2010;42: 19–42.

[26] Christen H. Convergence and divergence in the Swiss German dialects. Folia Linguistica. 1988;32: 53–67.

[27] Labov W. Sociolinguistic patterns. Philadelphia: University of Pennsylvania Press; 1979.

[28] Christen H, Glaser E, Friedli M. Kleiner Sprachatlas der Deutschen Schweiz. 5th ed Frauenfeld/Stuttgart/Wien: Huber; 2013.

[29] Hennig B. Kleines Mittelhochdeutsches Wörterbuch. 4th ed Tübingen: Niemeyer; 2001.

[30] BFS = Bundesamt für Statistik. Amtliches Gemeindeverzeichnis der Schweiz; 2012. Available: http://www.bfs.admin.ch/. Accessed 2014 Jan 13.

[31] Christen H. Ein Dialektmarker auf Erfolgskurs: Die /l/-Vokalisierung in der deutschsprachigen Schweiz. Zeitschrift für Dialektologie und Linguistik. 2001;1: 16–26.

[32] Leemann A, Kolly MJ, Werlen I, Britain D, Studer-Joho D. The diffusion of /l/-vocalization in Swiss German. Language Variation and Change. 2014;26: 191–218.

[33] Schilling N. Sociolinguistic fieldwork. Cambridge: CUP; 2013.

[34] Eckert P. Elephants in the room. Journal of Sociolinguistics. 2003;7: 392–397.

[35] Bucholtz M. Sociolinguistic nostalgia and the authentication of identity. Journal of Sociolinguistics. 2003;7: 398–416.

[36] Britain D. One foot in the grave?: Dialect death, dialect contact and dialect birth in England. International Journal of the Sociology of Language. 2009;196/197: 121–155.

[37] Milroy L, Gordon M. Sociolinguistics: Method and interpretation. Oxford: Blackwell; 2003.

[38] Labov W, Ash S, Boberg C. The atlas of North American English. Berlin: Mouton de Gruyter; 2005.

[39] Chambers JK. An introduction to dialect topography. English World-Wide. 1994;15: 35–53.

[40] Eskenazi M. An overview In: Eskenazi M, Levow GA, Meng H, Parent G, Suendermann D, editors. Crowdsourcing for speech processing: Applications to data collection, transcription and assessment. Hoboken, NJ: John Wiley & Sons; 1991 pp. 1–7.

[41] Lötscher A. Schweizerdeutsch: Geschichte, Dialekt, Gebrauch. Frauenfeld: Huber; 1983.

[42] Leemann A, Siebenhaar B. Perception of dialectal prosody. Proceedings of Interspeech 2008, Brisbane; 2008: 524–527.

[43] Guntern M. Erkennen von Dialekten anhand von gesprochenem Schweizerhochdeutsch. Zeitschrift für Dialektologie und Linguistik. 2011;78: 155–187.

[44] Clopper C, Pisoni D. Perception of dialect variation In: Pisoni D, Remez R, editors. The Handbook of Speech Perception. Oxford: Blackwell; 2005 pp. 313–337.

[45] Kehrein R, Lameli A, Purschke C. Stimuluseffekte und Sprachraumkonzepte In: Anders C, Hundt M, Lasch A, editors. Perceptual Dialectology. Neue Wege der Dialektologie. Berlin/New York: de Gruyter; 2010 pp. 351–384.

[46] Wolfensberger H. Mundartwandel im 20. Jahrhundert. Dargestellt an Ausschnitten aus dem Sprachleben der Gemeinde Stäfa. Frauenfeld: Huber; 1967.

[47] Trudgill P. Dialects in contact. Oxford: Blackwell; 1986.

[48] Stuart-Smith J, Pryce G, Timmins C, Gunter B. Television can also be a factor in language change: Evidence from an urban dialect. Language. 2013;89: 501–536.

[49] Sayers D. The mediated innovation model: A framework for researching media influence in language change. Journal of Sociolinguistics. 2014;18: 185–212.

[50] Chambers J. Dialect acquisition. Language. 1992;68: 673–705.

[51] Britain D. Supralocal regional dialect levelling In: Llamas C, Watt D, editors. Language and identities. Edinburgh: Edinburgh University Press; 2010 pp. 193–204.

[52] Trudgill P. On dialect. Oxford: Blackwell; 1983.

[53] Britain D. Between North and South: The Fenland In: Hickey R, editor. Researching Northern English. Amsterdam: Benjamins; 2015 pp. 417–436.

[54] Chambers J, Trudgill P. Dialectology. Cambridge: Cambridge University Press; 1998.

[55] Hotzenköcherle R. Die Sprachlandschaften der deutschen Schweiz. Bigler R, Schläpfer R, editors. Aarau: Sauerländer; 1984.

[56] Brabham DC. Crowdsourcing the public participation process for planning projects. Planning Theory. 2009;8(3): 242–262.

[57] Labov W. When intuitions fail In: Chicago Linguistic Society, editor. Papers from the parasession on theory and data in linguistics. Chicago: Chicago Linguistic Society; 1996 pp. 77–106.

[58] Laugwitz B. A web-experiment on colour harmony principles applied to computer user interface design In: Reips UD, Bosnjak M, editors. Dimensions of Internet science. Lengerich: Pabst Science Publishers; 2001 pp. 131–145.

[59] Cooke M, Barker J, Garcia Lecumberri ML. Crowdsourcing in speech perception In: Eskenazi M, Levow GA, Meng H, Parent G, Suendermann D, editors. Crowdsourcing for speech processing: Applications to data collection, transcription and assessment. Hoboken, NJ: John Wiley & Sons; 2013 pp. 137–172.

[60] Birnbaum MH. Human research and data collection via the Internet. Annu. Rev. Psychol. 2004;55: 803–832. 14744235 10.1146/annurev.psych.55.090902.141601

[61] Reips UD. Standards for Internet-based experimenting. Experimental Psychology. 2002;49: 243–256. 12455331 10.1026//1618-3169.49.4.243

[62] McGraw I. Collecting speech from crowds In: Eskenazi M, Levow GA, Meng H, Parent G, Suendermann D, editors. Crowdsourcing for speech processing: Applications to data collection, transcription and assessment. Hoboken, NJ: John Wiley & Sons; 2013 pp. 38–71.

[63] Bry F, Kneissl F, Krefeld T, Lücke S, Wieser C. Crowdsourcing for a geographical and social mapping of Italian dialects In: Proceedings of the 2nd International Workshop on Social Media for Crowdsourcing and Human Computation at ACM Web Science (SoHuman); 2013 pp. 4–7.

[64] Godwin-Jones R. Emerging technologies: Mobile apps for language learning. Language Learning & Technology. 2011;15: 2–11.

[65] Juska-Bacher B, Biemann C, Quasthoff U. Webbasierte linguistische Forschung: Möglichkeiten und Begrenzungen beim Umgang mit Massendaten. Linguistik online. 2012;61: 7–29.

[66] Vaux B, Leemann A, Moran S, Grimm S, Robert S, Zakharko T, et al. US Dialect App; 2014. Available: https://itunes.apple.com/us/app/us-dialect-app/id941252697?mt=8. Accessed 6 Nov 2015.

[67] Brupbacher M, Elmer C, Grossenbacher T, Leemann A, Kolly M-J, Grimm S, et al. Grüezi, Moin, Servus; 2015. Available: http://sprachatlas.spiegel.de/. Accessed 6 Nov 2015.

